# Reports of unintended consequences of financial incentives to improve management of hypertension

**DOI:** 10.1371/journal.pone.0184856

**Published:** 2017-09-21

**Authors:** Sylvia J. Hysong, Richard SoRelle, Kristen Broussard Smitham, Laura A. Petersen

**Affiliations:** 1 Houston VA HSR&D Center for Innovations in Quality, Effectiveness and Safety, Michael E. DeBakey VA Medical Center, Houston, Texas, United States of America; 2 Department of Medicine, Baylor College of Medicine, Houston, Texas, United States of America; 3 VISN 4 Center for Evaluation of PACT (CEPACT), Corporal Michael J. Crescenz VA Medical Center, Philadelphia, Pennsylvania, United States of America; Université Versailles Saint-Quentin en Yvelines, FRANCE

## Abstract

**Background:**

Given the increase in financial-incentive programs nationwide, many physicians and physician groups are concerned about potential unintended consequences of providing financial incentives to improve quality of care. However, few studies examine whether actual unintended consequences result from providing financial incentives to physicians. We sought to document the extent to which the unintended consequences discussed in the literature were observable in a randomized clinical trial (RCT) of financial incentives.

**Methods:**

We conducted a qualitative observational study nested within a larger RCT of financial incentives to improve hypertension care. We conducted 30-minute telephone interviews with primary care personnel at facilities participating in the RCT housed at12 geographically dispersed Veterans Affairs Medical Centers nationwide. Participants answered questions about unintended effects, clinic team dynamics, organizational impact on care delivery, study participation. We employed a blend of inductive and deductive qualitative techniques for analysis.

**Participants:**

Sixty-five participants were recruited from RCT enrollees and personnel not enrolled in the larger RCT, plus one primary care leader per site.

**Results:**

Emergent themes included possible patient harm, emphasis on documentation over improving care, reduced professional morale, and positive spillover. All discussions of unintended consequences involving patient harm were only concerns, not actual events. Several unintended consequences concerned ancillary initiatives for quality improvement (e.g., practice guidelines and performance measurement systems) rather than financial incentives.

**Conclusions:**

Many unintended consequences of financial incentives noted were either only concerns or attributable to ancillary quality-improvement initiatives. Actual unintended consequences included improved documentation of care without necessarily improving actual care, and positive unintended consequences.

**Trial registration:**

Clinicaltrials.gov Identifier: NCT00302718

## Introduction

To align payment systems with higher-quality care, many health-care organizations have turned to pay-for-performance programs in which physicians receive financial incentives for meeting quality targets [1]. Given the increase in financial-incentive programs nationwide, many physicians and physician groups are concerned about unintended consequences. This is not surprising, given research pointing to unintended consequences of related quality-improvement activities, such as performance-measurement systems [2].

Concerns regarding financial incentive programs include decreased attention to non-incentivized clinical activities, declining physician professionalism, gaming behaviors (i.e., increasing performance-measure scores without increasing care quality), declines in physician morale and job satisfaction, damage to ongoing patient-physician relationships, increased health-care disparities, and stifling of innovation [3–10]. However, few have studied actual unintended consequences of financial incentives—one review found four financial incentive studies addressing this issue. ^3^ More recently, a randomized controlled trial (RCT) of financial incentives for hypertension management significantly improved care without unintended consequences, such as hypotension [4].

Our PubMed search identified only eight studies since Petersen and colleagues’ 2006 review evaluating whether certain unintended consequences resulted from financial-incentive programs. Some sought to determine unintended consequences for physicians in family practice, [5–7] on patients’ experiences with their primary care providers [8,9], and on non-incentivized clinical activities [10]. The two studies examining unintended consequences on patient experiences found negative effects on patient satisfaction [8,9] and continuity of care [9], although access to urgent care improved for patients with chronic illnesses [9]. In research examining physicians or practices, introducing financial-incentive programs often impacted the nature of the office in multiple ways, including reliance on computerized medical records for data collection, quality targets’ distracting physicians from the patients’ agenda, threats to disenroll overly complex patients or greater emphasis on securing patient compliance, and decreased physician autonomy [5,6]. Finally, one study [10] found that nonincentivized activities improved more slowly than incentivized activities; additionally, incentivized activities improved more slowly than they had before introduction of the incentive.

Most studies were not specifically designed to study unintended consequences of financial incentives; and most occurred in the United Kingdom after the National Health Service implemented its financial-incentives program. The UK studies generally did not include a concurrent control group. Our study addresses some gaps in the literature. Nested within an RCT of financial incentives, it examines whether RCT participants and concurrent controls reported unintended consequences of receiving financial incentives.

## Method

Details of the cluster RCT, which evaluates the impact of financial incentives on primary care providers’ adherence to hypertension guidelines, appear elsewhere [11]; we summarize selected components here. Primary care clinics within 12 geographically diverse Veterans Affairs medical centers were randomly assigned to one of four groups: physician-level incentive + audit and feedback; practice-level incentive + audit and feedback; combined incentives + audit and feedback (combined incentive); audit and feedback only (control). The study was approved by the Baylor College of Medicine Institutional Review Board (IRB), the principal investigator’s home institution (protocol #H-17777) and the institutional review boards of all participating institutions [12].S1–S7 Files provide details of all IRB-related approvals, protocol and findings of the cluster RCT, and COREQ reporting guidelines checklist for the present study. All participants provided written informed consent.

### Setting

The Veterans Health Administration (VHA) is ideal for conducting financial-incentives research because of its size and integrated structure [13]. It can be construed as a single-payer health-care system, eliminating payer variations as a potential source of confounding. VHA physicians are salaried employees, making standardized incentive delivery possible across sites. Integrated electronic medical records minimize differences in documentation and data processing and facilitate centralized chart review for outcomes data collection. Finally, patients are assigned to physicians as they enroll in the VHA system, virtually eliminating risk selection as a potential unintended consequence. In essence the VHA represents a “best case scenario”–if financial incentives cannot be successfully implemented there, it would be far more difficult to do so in the private sector, where both practice and payers are fragmented.

### RCT intervention components

#### Provider education

Participants received a standardized, web-based presentation summarizing guidelines from the *Seventh Report of the Joint National Committee on Prevention*, *Detection*, *Evaluation*, *and Treatment of High Blood Pressure* [14] and educating about their study-group assignment and study performance measures. Participants also received summary pocket cards and web links to this pocket card and other educational resources.

#### Financial incentives

Participants in intervention groups received payments commensurate with their guideline adherence approximately every 4 months over 20 months. Incentive payments rewarded chart-documented use of guideline-recommended antihypertensive medications, BP control, and guideline-recommended responses to uncontrolled BP [11]. In the individual-incentive group, each physician received a direct payment, based on his/her individual adherence. In the practice-level group, a payment based on the collective performance of participating physicians in the practice was divided equally among all practice members (both physician and nonphysician). In the combined group, each physician received a direct payment, based on individual adherence; additionally, a payment based on the collective adherence of physicians in the practice was divided equally among all practice members.

#### Audit and feedback

Audit and feedback reports (S8 File) were delivered to participants in all groups approximately every 4 months for 5 consecutive periods via a website. Reports were designed using Feedback Intervention Theory [15], paying attention to feedback characteristics that improve feedback effectiveness in health-care settings [16,17].

### Site selection

Study sites were selected based on characteristics expected to be associated with effectiveness of financial incentives: teaching status, geographic location, participation in the Antihypertensive and Lipid-Lowering treatment to prevent Heart Attack Trial (ALLHAT [18], a large trial of medications for treating hypertension that included intensive education regarding hypertension control and use of evidence-based hypertension treatment), and degree of clinic geographic proximity within the primary care setting at each study site (Table 1). Sites were randomized to one of the previously mentioned groups, using constrained randomization.

**Table 1:** Study-site characteristics.

VA Hospital	City, State	Teaching facility*	US Census Division	ALLHAT study site	Primary care geographic integration†
VA Boston HCS	Boston, MA	X	New England		X
Providence VAMC	Providence, RI	X	New England	X	X
VA Connecticut HCS	Newington, CT		New England		X
Charlie Norwood VAMC	Augusta, GA	X	South Atlantic	X	X
Ralph H. Johnson VAMC	Charleston, SC	X	South Atlantic	X	X
Birmingham VAMC	Birmingham, AL	X	E. South Central		X
Aleda E. Lutz VAMC	Saginaw, MI		E. North Central		
John D. Dingell VAMC	Detroit, MI	X	E. North Central	X	X
G.V. (Sonny) Montgomery VAMC	Jackson, MS	X	E. South Central	X	
Michael E. DeBakey VAMC	Houston, TX	X	W. South Central	X	
Oklahoma City VAMC	Oklahoma City, OK	X	W. South Central	X	X
Minneapolis VAMC	Minneapolis, MN	X	W. North Central	X	

ALLHAT = Antihypertensive and Lipid-Lowering Treatment to Prevent Heart Attack Trial; HCS = healthcare system; US = United States;

VA = Veterans Administration; VAMC = Veterans Affairs Medical Center

*Designated as a teaching facility if the facility was either listed in the Association of American Medical College's Council of Teaching Hospitals directory or if the American Medical Association's Fellowship and Residency Electronic Interactive Database database listed the VA facility as having a “major” affiliation with a medical school.

^†^Designated as geographically integrated if the primary care clinic layout was amenable to group cohesion (*e*.*g*., the primary care clinic offices located on the same floor at the study site).

Reprinted with permission from Petersen et al. Design, rationale, and baseline characteristics of a cluster randomized controlled trial of pay for performance for hypertension treatment: study protocol. Implement Sci. 2011;6: 114.[11] BioMed Central is the original publisher.

**Fig 1:**
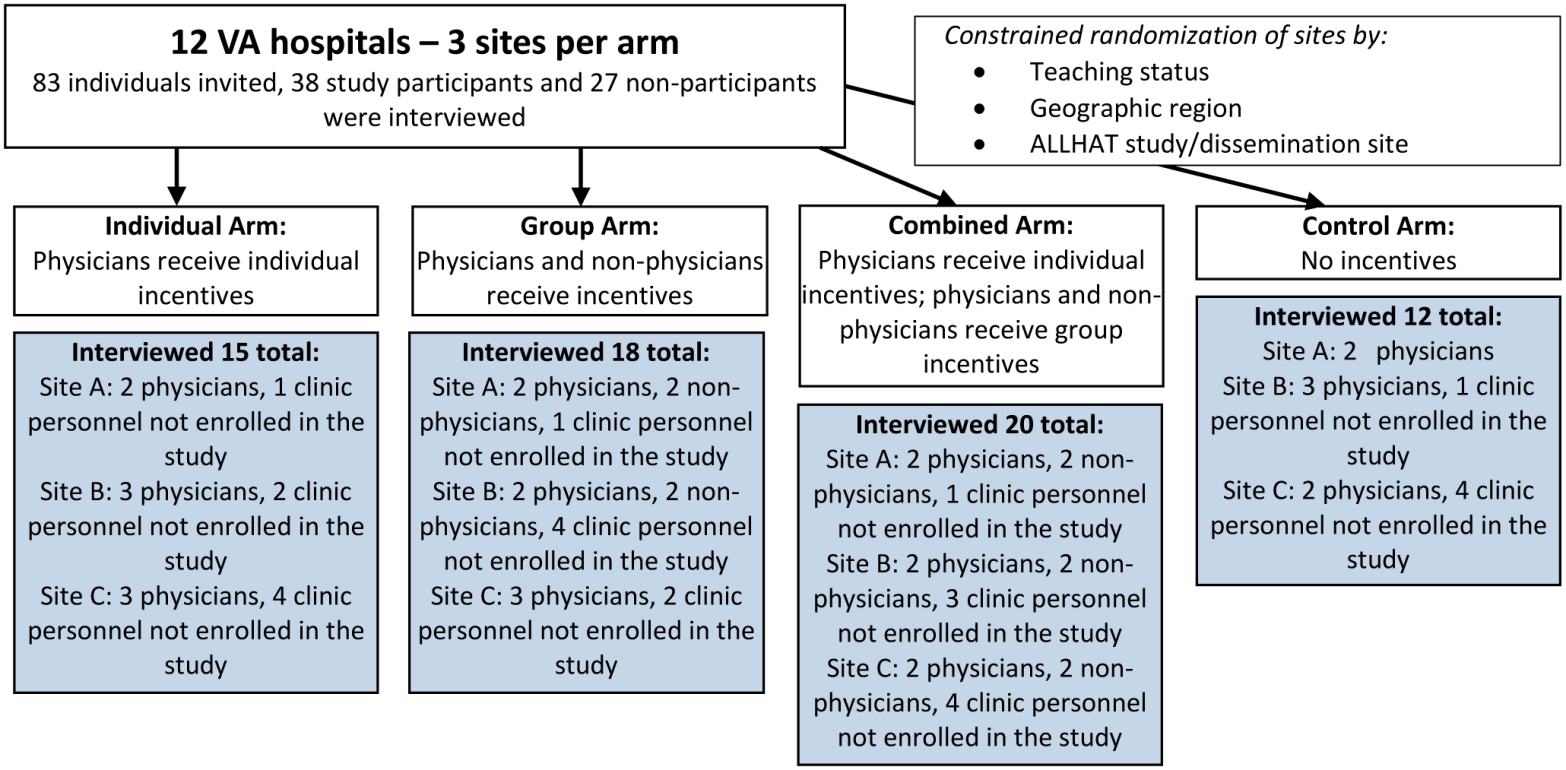
Randomized controlled trial design.

We randomly selected 3 physician participants from each site receiving individual incentives and combined practice and individual incentives, and from each control site; at sites receiving practice-level incentives, we randomly selected 2 physician and 2 nonphysician participants. Additionally, at each site we interviewed 4 clinic members not enrolled in the study, plus 1 individual in Primary Care practice leadership. All personnel must have held their current position for at least 6 months during the intervention period to be eligible for interview. Fig 1 illustrates participant breakdown by study arm.

### Procedures

Participants were interviewed individually via telephone for 30 minutes by a trained research assistant. Interviews were audio-recorded with the participant’s consent and transcribed for analysis; if the participant declined to be recorded, a trained note-taker typed notes. Participants answered questions about unintended effects, team dynamics in the clinic, organizational changes and their impact on care delivery, and participation in the study. S9 File presents the interview guide used.

### Analysis

We employed a blend of inductive [19,20] and deductive [21] qualitative techniques, as recommended by Hsieh and colleagues [22], to analyze interviews, detailed below.

#### Analysts’ background

Data were coded and analyzed by a master’s-level industrial/organizational (I/O) psychologist, and a research assistant with master’s-level training in sociology and training in qualitative methods, both supervised by a doctoral-level I/O psychologist with experience and expertise in the qualitative methods employed.

#### Open coding

Open coding names, categorizes, and describes phenomena in interview transcripts. The same research assistants who conducted interviews conducted open coding. All interviews were coded (as opposed to relying on thematic saturation as a stopping criterion).

Guided by the review by Petersen et al. [11], coders received an *a priori* list of unintended consequences and their definitions, designed to capture relevant constructs of interest. Using this list, coders selected relevant interview passages indicative of a given phenomenon and assigned them a descriptive label. Notably absent from the *a priori* list is “cherry-picking,” as the VHA system assigns patients to primary care providers [22,23]. Coders reviewed transcripts for instances of constructs in the research questions and identified new unintended consequences not in the existing list.

Each research assistant served as primary coder for half of interview transcripts and as secondary coder for the remaining half. Primary coders assigned relevant codes to transcripts; secondary coders reviewed primary coders’ codings and concurred or disagreed. All primary coding was completed before beginning secondary coding. Disagreements were resolved by consensus.

#### Ensuring confirmability and trustworthiness

The doctoral-level I/O psychologist (who deliberately conducted no open coding) served as tiebreaker when the 2 original coders could not agree. Assumptions and impressions generated during interview coding were documented simultaneously with the originally planned coding as interviews were coded. Coders documented their rationale for code assignment while coding and expressly sought out contrasting viewpoints. Consensus code assignments and their rationales were also documented in vivo. These materials were constantly referenced during coding to check for bias. Together, these strategies helped minimize potential biases resulting from differences in experiences of interviewers and coders and maximize analyses’ confirmability and trustworthiness.

#### Axial coding and thematic analysis

**Fig 2:**
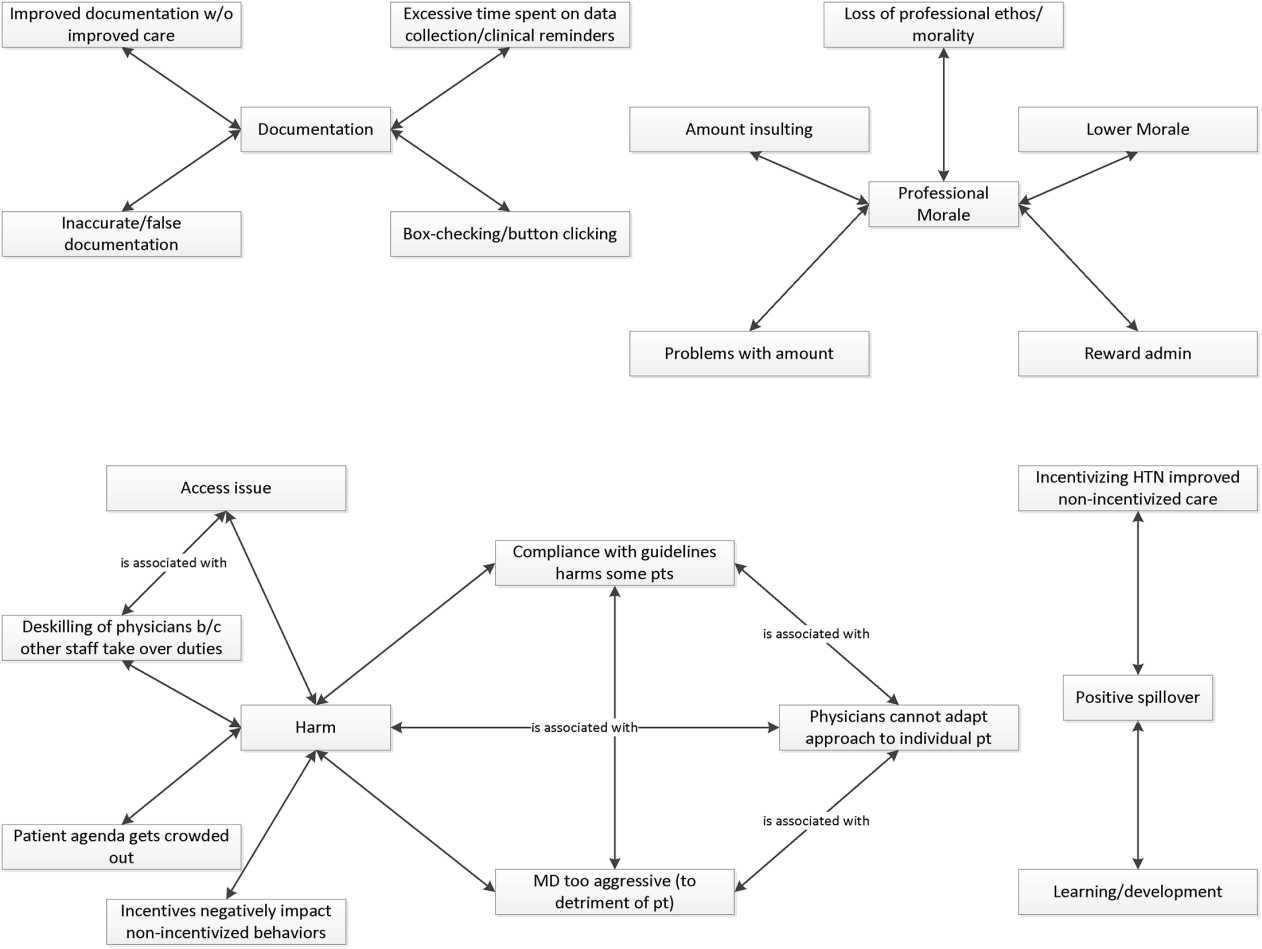
Network diagram of codes and organizing themes.

Next, the research team organized the code taxonomy into salient themes (also by consensus), considering each code’s groundedness (how often participants mentioned it), co-occurrence with other codes, and presence in single vs. multiple interviews. The prevalence of these themes was then compared by study group and participant type. S10 File presents the final set of codes and their definitions, organized by theme. Fig 2 presents the codes visually as they relate to the themes and as they co-occur with each other.

#### Clarifying the boundaries of unintended consequences

During axial coding we coded quotations that referred only to concerns or unintended consequences of ancillary phenomena, such as the VHA national performance-measurement system, unrelated to the study.

Additionally, during the study, the VHA introduced a bonus pay system for physicians, based on clinical-performance measures[24]. Unintended consequences of this incentive were coded separately, as were unintended consequences of any other quality-improvement initiatives emergent from the data.

## Results

### Types of unintended consequences

Participants endorsed 11 of 17 unintended consequences from the literature, plus 7 additional unintended consequences. These 18 were grouped into 9 broader categories and organized into themes. Four themes emerged from issues raised by the 65 invited participants (38 trial participants plus 27 clinic personnel who did not participate): possible patient harm, emphasis on documentation over improving care, reduced professional morale, and positive spillover. Table 2 shows brief descriptions and illustrative quotations for each theme.

**Table 2:** Theme descriptions and illustrative quotations.

Theme Name and Description	Illustrative Quotations
**1. Patient Harm:** experience that measuring clinical performance via strict adherence to guideline-recommended care may cause patient harm	***A. Reduced Flexibility to individualize care (****Physician, individual-incentive site B****):*** *I would actually be concerned that [incentivizing guideline-recommended care] could do the opposite, that people would actually, in this case, start to prescribe more thiazides in situations where other drugs would be first line because of a financial incentive to use that medication*. ***B. Pressure to treat incentivized condition (****Physician, practice-level incentive site A)***:** *You know, it’s not like I do any studies analysis. Just, by encountering patients all these years, I perceive we do have numerous patient we have to deal with, with too aggressive to bring their blood pressure down; then because in this, uh, specific encounter the patient group we deal with lot these elderly. Most patient probably is over 60, or 70, 80. Then, you know, once patient get this age, if you, they had other comorbidity, you bring the blood pressure too low they, they could easily fall because they already had the problem with arthritis or balance or dementia and the physically they so weak, then you too aggressive, push it down, then they fall, they broken hips or they cannot function well*.
**2. Documentation**: spending too much time on documentation so that performance measures could be calculated, or efforts to document more effectively without actually improving care	***P:*** *Well, I think the only major difference is the documentation piece because I realized a lot of the stuff I do wasn’t documented adequately so it wasn’t reflecting the quality of my care. So if I don’t go in after re-checking the BP then it won’t reflect my work, even if I put it in my note. So now I know how to play the game so that when they do their data mining, it’s showing up in there. **I:** Do you think it’s useful to document in that way? **P:** No, the important thing is that the patient’s BP is at goal, not the way that data mining is occurring. The documentation doesn’t reflect the way that the care is being provided*. -- *Physician, individual-incentive site B*
**3. Professional Morale**: potential for financial incentives to impact morale or attitudes of clinical staff and leaders	***A*. *Inequity over who receives financial incentives*** *(Physician*, *combined-incentive site A)***:** *Well, I know the nurse practitioners are totally unhappy about the fact that they are not entitled to a [VA] bonus every year. I mean, what gives them the incentive; and, truthfully, I think that’s part of the reason there’s a disparity in quality because what’s their incentive? They don’t get an incentive. At least you could argue—for me it doesn’t make a difference.… but I can imagine that there may be some people for whom it would make a difference, and the nurse practitioners are totally excluded from this; and I don't know why that is, and it’s not my battle to fight, but I totally empathize with them. I think if they’re doing the same job I'm doing then they should be entitled to the bonus as well, but that’s just my opinion, and I have no power. I am just one of the worker bees*. ***B*. *Resentment against leadership receiving incentives*** *(Physician*, *individual-incentive site B)*: *We've known about it for a long time, that the people that are making us do all this clicking get a bonus if we click adequately. So we used to talk about it. About a year ago we sat around, we were so pissed off about it, we said we’re all going to stop clicking. Just that we were so angry with our bosses that were making us do this, these administrators that do nothing but take up space. They're not seeing patients, they're not taking care of patients, they don’t know what it is to take care of patients, but they're making us do all this stuff. We said, fine, we’ll just stop clicking; let them lose some money*. ***C*. *Concern that motivations will shift towards incentives over best care (****Physician*, *control site A)***:** *But I think, overall, that financial-incentive motivators can detract from the ethics of what you're trying to do. People go into medicine because they're interested in it and because they care about the welfare of other people, and when you start reducing it to a paycheck, then that’s how people start to think, and it could create animosity, too*. ***D*. *Negative case*: *incentives not affecting professionalism*** *(Physician*, *combined-incentive site A)*: *I recently had a patient that I referred to the hypertensive clinic because he was on, like, four or five different drugs; and I still couldn’t get him controlled, and the doctor in the hypertension clinic … said you should not try to get this patient to goal. There has been a recent study that shows that 130/80 for a diabetic has increased mortality and morbidity and when the new JNC guidelines come out it’s gonna be totally different. So he says forget the reminders, because we get clinical reminders. It’ll pop up. Regardless of your study, if it knows that this patient is a diabetic and the recorded blood pressure when they walked in was above 130/80, I'm gonna get a reminder at the end of my note that says this blood pressure is above goal, what are you gonna do about it, and that’s the long and short of it. In any case, he was saying forget the reminder, don’t try to satisfy it, do what’s right for your patient because you don’t want to cause increased mortality and morbidity*.
**4. Positive Spillover:** financial incentives having positive unintended consequences	***A*. *Incentives inspired provider to educate themselves better*** *(RN*, *combined-incentive site C)***:** **P:** *Well, I've just been doing some further research, just reading different articles that come across, looking at different things that had impact, the different gender, across the gender, of course, the age, their diets and their activities, stressors, where they live, how they're living, just reading up, doing a little bit more research, being more aware and more knowledgeable on how to treat it.…*. ***I:*** *Do you attribute that to the study*? ***P:*** *I do so, I really do because reading the different little information I have come across here, and then I have a husband that’s hypertensive at home; it helps me to help him manage his high blood pressure at home. So this study has been very, very informational and very supportive towards my knowledge base in knowing how to manage hypertension a little bit better*. ***B*. *Associating incentives with increased professionalism (****NP*, *practice-level incentive site B)* *NPs don’t get financial reimbursement, only the physicians do, and this was the first time I actually felt like I was being treated as an equal, and that went a long way with me, so I really did my best during the study*.

Table 2. Themes describing reported unintended consequences and concerns, and illustrative quotations.

#### Patient harm

This theme refers to participants' experience that measuring clinical performance via strict adherence to guideline-recommended care may cause patient harm, for example, by not allowing physicians to consider the “whole patient.” Participants from all groups expressed concern that financial incentives may reward behaviors based on guidelines that sometimes do not allow a physician the flexibility to individualize care, thereby leading to patient harm (see Table 2, quotation 1A). Participants in the practice-level incentive group also noted that sometimes pressure to treat the incentivized condition can result in treating patients too aggressively, which could also harm (Table 2, quotation 1B).

#### Documentation

This theme concerns incidents when documentation requirements appear to add little value to and/or sometimes hinder quality of care. Although several issues fit into this theme, the focal concern was simply spending too much time on documentation so that performance measures could be calculated. A somewhat more frequently mentioned phenomenon was providers who simply documented more effectively care they were already providing, without actually improving care. Quotation 2 in Table 2 illustrates both phenomena.

#### Professional morale

This theme concerned incidents in which performance measures and their corresponding financial incentives impacted morale or attitude of clinical staff and leaders, particularly the question of who receives financial incentives, relative to who does the work. In some cases—particularly when referring to the VHA’s bonus pay system—participants noted that nonphysicians resented physicians’ being the only clinicians receiving financial incentives (Table 2, quotation 3A). A variant of this theme is resentment against facility leadership’s receiving incentives, based upon provider performance (Table 2, quotation 3B). In both examples, participants discussed impacts of ancillary VHA initiatives, such as a bonus pay system for administrators only, not the study incentive.

A final manifestation of this theme was the concern that financial incentives would cause physicians’ motivations to shift from providing the best care to providing whatever care is incentivized (Table 2, quotation 3C). However, although several participants raised this issue, in every case it was a concern, discussed primarily by control-group participants. This was never discussed by any incentive-group study participants as something that they felt was happening (Table 2, quotation 3D).

#### Positive spillover

Finally, we encountered statements suggesting the presence of positive unintended consequences. For example, participants reported incidents when being knowledgeable about hypertension guideline-recommended care, performance measures, and incentives resulted in a holistic or whole-person approach to a patient’s care/health, as with this participant, who further educated herself on hypertension care (Table 2, quotation 4A). We also encountered reports of participants associating incentives with increased professionalism (Table 2, quotation 4B).

### Separating the reality from the concerns

One important part of this analysis was differentiating reports of actual events resulting in unintended consequences from concerns about financial incentives. Thus, the research team reviewed the text of each specific quotation and its surrounding context to make a determination about this. Most instances of the documentation theme and all instances of the positive unintended consequences theme were found to be actual events rather than simply concerns (see Table 3). All discussions of unintended consequences involving harm to patients (the harm theme) were only concerns, not actual events. A chart audit of the larger RCT found no differences across study arms in cases of hypotension, which would have been an expected though unintended consequence of our financial-incentives study.^4^ Additionally, a common concern in the literature is that incentives will override professional standards (“loss of professional ethos/morality”). Few study participants discussed this issue related to their observations of other physicians or other clinical staff; none provided instances in which he/she felt financial incentives threatened his/her own moral judgment, though, admittedly, participants may have avoided disclosing this socially undesirable behavior. In fact, 1 nurse practitioner participant noted that the incentives enhanced self-perceived professionalism.

**Table 3:** Presence and absence of study-related codes organized by theme and ancillary causes.

	ANCILLARY CAUSES							
Code	Clinical Reminder		Guidelines		Performance Measurement		Financial Incentives	
*Study arm →*	*Control*	*Incentive*	*Control*	*Incentive*	*Control*	*Incentive*	*Control*	*Incentive*
**Documentation**								
Improved documentation without improved care								
Excessive time spent on data collection/clinical reminders								
**Harm**								
Reduced Flexibility to individualize care								
Pressure to treat incentivized condition to detriment of patient								
Incentives negatively impact nonincentivized behaviors								
**Positive Spillover**								
Improved morale								
Learning/Development								
Incentivizing HTN improved nonincentivized behaviors								
**Professional Morale**								
Loss of professional ethos/morality								

Note. Cells shaded in light gray indicate concerns; cells in dark gray indicate actual occurrences of unintended consequences. Cells in white indicate the consequence was not observed in that group.

HTN = hypertension

### Financial incentives versus ancillary issues

It was also important to determine whether unintended consequences reported were actually created by introducing financial-incentive payment systems versus other quality-assessment or improvement initiatives. For example, some examples of patient harm presented earlier did not reflect confirmed instances of the financial incentive’s overriding participants’ clinical judgment but were concerns that financial incentives might cause other clinicians to follow guideline-recommended care too rigidly or treat hypertension too aggressively, implying that their concern was not with the financial incentive per se, but rather with guidelines they are asked to follow. Thus, the research team reviewed the text of each specific quotation and its surrounding context to determine whether the concern was indeed with the study’s financial incentives or with ancillary initiatives.

We found that several unintended consequences raised related more to implementation of practice guidelines, clinical-reminder systems, and performance-measurement systems in general (see Table 3). In particular, when participants discussed unintended consequences within the harm theme, they were often more likely to be referring to the VHA’s clinical-reminder system (unrelated to this study).

In the documentation theme, participants who discussed problems with excessive data collection referred mostly to the VHA’s clinical-reminder system; the other major concern, that financial incentives would result in improved documentation without genuine improvement in care, was related only to financial incentives. We note that this unintended consequence is less related to *provision* of financial incentives than to a consequence of the infrastructure needed to implement performance measures and incentives, something the VHA implemented separately from our study. The “loss of professional ethos/morality” code, under the professional morale theme, and all codes in the positive-spillover theme were related to concerns about, and not actual consequences of, financial incentives.

## Discussion

Four themes emerged from participants’ discussions of unintended consequences of financial incentives: harm to patients, documentation, professionalism issues, and positive unintended effects. Many, particularly in the harm theme, were concerns rather than actual instances in which clinical judgment was overpowered by a monetary incentive. Some reported unintended consequences were actually positive, rather than negative, such as 1 participant saying that he/she felt that the focus on hypertension helped him/her to focus on other conditions, such as diabetes and hyperlipidemia, as well. Finally, several unintended consequences were traceable to quality-improvement interventions, such as the VHA’s clinical-reminder system or clinical-practice guidelines, rather than to the introduction of financial incentives for health-care quality.

Many unintended consequences were consistent with the literature. Additionally, those under the harm theme were very similar to the theme of inappropriate clinical care found to be an unintended consequence of performance measurement.[25] However, our finding that many unintended consequences were concerns rather than real events is a unique contribution to the literature; similarly, many consequences raised were related more to the data-collection, clinical-reminder, and performance-measurement systems than financial-incentive programs or the study itself. Kizer and Jha call attention to the VHA’s large and unfocused performance-measurement program as 1 cause of ongoing concern in delivery of safe and effective care.[26] While the VHA performance-measurement program of the late 1990s improved quality and accountability, the current program contains hundreds more measures of performance with varying degrees of clinical importance. The authors suggest refocusing the program and narrowing measures to those most important to patients and clinicians. Of studies we reviewed, none specifically made these distinctions in their findings. Future work should clearly delineate effects of quality assessment vs. financial-incentive programs.

Finally, some participants listed positive unintended effects of the financial incentives, an issue absent in literature we reviewed. This presents a unique opportunity to research ways in which financial incentives, whether by themselves or combined with other interventions, can enhance the clinician work experience beyond the original intent.

### Limitations

This study had several limitations. First, during our study, the VHA implemented its own yearly bonus pay program consisting of up to 15% of physicians’ salaries for generalized performance (i.e., it was not clear which performance metrics specifically drove a given physician’s pay). If it was not implemented successfully at their facility, participants’ attitudes towards financial incentives and, consequently, their interview responses, may have been influenced independent of the study.

Second, participants were often more likely to discuss effects of the VHA’s bonus pay program than the study incentive, implying that the VHA’s incentive was more salient than the study incentive. Therefore, we might have noted more unintended consequences, if we were to have offered a larger study incentive. However, overall findings of the main study [25] and the presence of positive unintended consequences argue for the effectiveness and salience of a relatively modest financial incentive, such as the one studied here.

Finally, we did not assess whether reported unintended consequences correlated with actual consequences; this was beyond the scope of our study and an excellent area for future work.

### Conclusions/Future directions

Many unintended consequences of financial incentives noted by participants were either only concerns or attributable to ancillary quality-improvement initiatives. Actual unintended consequences of financial incentives observed consisted largely of (a) *positive unintended consequences*, such as increased focus on conditions, interest in additional education concerning hypertension care, and a feeling of greater professionalism for nurses; and (b) *improved documentation of care without necessarily improving the care provided*. Nevertheless, incentives may intensify other problems caused by ancillary systems. Future studies should determine how incentives and ancillary systems interact to produce clinical outcomes, as well as how to optimize their design to maximize desired clinical outcomes while minimizing negative unintended consequences.

## Supporting information

S1 File Parent study protocol: Petersen et al. 2011 implementation science. (PDF)

S2 File Parent study main findings: Petersen et al. 2013 JAMA. (PDF)

S3 File Permission from Biomed Central to reproduce Table 1 from Petersen et al. 2011. (PDF)

S4 File COREQ guidelines checklist. (DOCX)

S5 File Original IRB approval. (PDF)

S6 File Most current IRB approval. (PDF)

S7 File Human protocol, interview language highlighted. (PDF)

S8 File Appendix A: Sample feedback report. (DOCX)

S9 File Appendix B: Interview question guide. (DOCX)

S10 File Appendix C: Codes and final code definitions. (DOCX)
