# Supplementary material for: Reports of unintended consequences of financial incentives to improve management of hypertension
Source: PLoS One. 2017 Sep 21;12(9):e0184856. doi: 10.1371/journal.pone.0184856 (PMC5608267; doi:10.1371/journal.pone.0184856)
Supplement: S2 File — (PDF) [file pone.0184856.s002.pdf]

Original Investigation

Effects of Individual Physician-Level and Practice-Level Financial Incentives on Hypertension Care
A Randomized Trial

Laura A. Petersen, MD, MPH; Kate Simpson, MPH; Kenneth Pietz, PhD; Tracy H. Urech, MPH; Sylvia J. Hysong, PhD; Jochen Profit, MD, MPH; Douglas A. Conrad, PhD; R. Adams Dudley, MD, MBA; LeChauncy D. Woodard, MD, MPH

IMPORTANCE Pay for performance is intended to align incentives to promote high-quality care, but results have been contradictory.

OBJECTIVE To test the effect of explicit financial incentives to reward guideline-recommended hypertension care.

DESIGN, SETTING, AND PARTICIPANTS Cluster randomized trial of 12 Veterans Affairs outpatient clinics with 5 performance periods and a 12-month washout that enrolled 83 primary care physicians and 42 nonphysician personnel (eg, nurses, pharmacists).

INTERVENTIONS Physician-level (individual) incentives, practice-level incentives, both, or none. Intervention participants received up to 5 payments every 4 months; all participants could access feedback reports.

MAIN OUTCOMES AND MEASURES Among a random sample, number of patients achieving guideline-recommended blood pressure thresholds or receiving an appropriate response to uncontrolled blood pressure, number of patients prescribed guideline-recommended medications, and number who developed hypotension.

RESULTS Mean (SD) total payments over the study were $4270 ($459), $2672 ($153), and $1648 ($248) for the combined, individual, and practice-level interventions, respectively. Change in blood pressure control or appropriate response to uncontrolled blood pressure compared with the control group was significantly greater only in the individual incentives group. Change in guideline-recommended medication use was not significant compared with the control group. The effect of the incentive was not sustained after a washout.

	Individual	Practice	Combined	None (Control)
**Blood Pressure Control or Appropriate Response to Uncontrolled Blood Pressure, % of Physicians' Patients (95% CI)**				
Baseline	75	80	79	86
Adjusted change from baseline	8.84 (4.20 to 11.80)	3.70 (0.24 to 7.68)	5.54 (1.92 to 9.52)	0.47 (−3.12 to 4.04)
Difference in change vs control	8.36 (2.40 to 13.00)	3.24 (−1.48 to 8.92)	5.08 (−0.04 to 10.56)	1 [Reference]
**Use of Guideline-Recommended Antihypertensive Medications, % of Physicians' Patients (95% CI)**				
Baseline	61	56	65	63
Adjusted change from baseline	9.07 (4.52 to 13.44)	4.98 (0.64 to 10.08)	7.26 (2.92 to 12.48)	4.35 (−0.28 to 9.28)
Difference in change vs control	4.72 (−1.44 to 10.92)	0.64 (−5.32 to 7.32)	2.92 (−2.76 to 9.64)	1 [Reference]

CONCLUSIONS AND RELEVANCE Individual financial incentives, but not practice-level or combined incentives, resulted in greater blood pressure control or appropriate response to uncontrolled blood pressure; none of the incentives resulted in greater use of guideline-recommended medications or increased incidence of hypotension compared with controls. Further research is needed on the factors that contributed to these findings.

TRIAL REGISTRATION clinicaltrials.gov Identifier: NCT00302718

JAMA. 2013;310(10):1042-1050. doi:10.1001/jama.2013.276303

Editorial page 1031

Author Video Interview at jama.com

Related article page 1051

Supplemental content at jama.com

Author Affiliations: Author affiliations are listed at the end of this article.

Corresponding Author: Laura A. Petersen, MD, MPH, Health Services Research and Development (152), Michael E. DeBakey Veterans Affairs Medical Center, 2002 Holcombe Blvd, Houston, TX 77030 (laura.petersen@va.gov).

Downloaded From: http://jama.jamanetwork.com/ by a Department of Veterans Affairs User on 01/22/2014

As part of the Affordable Care Act, the US government has introduced pay for performance to all hospitals paid by Medicare nationwide.[1] The New York City Health and Hospitals Corporation recently announced a performance pay plan for physicians.[2] These and other value-based purchasing systems are intended to align incentives to promote high-quality health care.[3] Evaluations of the effectiveness of pay-for-performance programs directed at hospitals have shown contradictory results.[3-5] The Premier Hospital Quality Incentive Demonstration showed an increase of up to 4.1 percentage points in process-quality measures during the first 2 years,[6] but these modest gains were not sustained.[7] Moreover, risk-adjusted mortality in the same program showed no improvement.[8]

In contrast, recent studies assessing outcomes of hospital pay-for-performance programs implemented in the United Kingdom on a wider scale, with larger bonuses and with different approaches to quality improvement, showed clinically significant mortality reductions.[9] Enhancing the face validity of these findings, these reductions were concentrated among hospitals that also showed the best process-measure performance.

Evaluations of incentives targeted at individual physicians and physician practice teams (ie, clinicians, nurses, and support staff who deliver health care) also show variability.[3-5] A Cochrane review of incentives to improve the quality of primary care found that 6 of 7 eligible studies showed a statistically significant positive effect, but the authors encouraged caution in interpreting findings because of design limitations and generalizability concerns.[4] Thus, many questions about pay for performance are unanswered.[4,5,10]

Given the implementation of the patient-centered medical home and models of accountable care,[11] the effects of financial arrangements that reward health care practice teams will become more interesting to payers and policy makers.[12] We are not aware of other multisite randomized trials of pay for performance directed at both physicians and practice teams. Therefore, we designed a cluster randomized controlled trial to test the effect of explicit financial incentives to individual physicians and practice teams for the delivery of guideline-recommended care for hypertension in the primary care setting.

Methods

Study Design and Randomization

Characteristics of the study hospitals and detailed trial methods were published elsewhere.[13] Research assistants at the Houston coordinating center enrolled a minimum of 5 full-time primary care physicians from 12 hospital-based primary care clinics in 5 Veterans Affairs (VA) Networks. Then, the clinics were randomized to 1 of 4 study groups: (1) physician-level (individual) incentives; (2) practice-level incentives; (3) physician-level plus practice-level (combined) incentives; and (4) no incentives (control). We cluster-randomized by hospital to avoid contamination of the intervention; all participants at a hospital belonged to the same intervention group.[14] Randomization was constrained on teaching status, geographic and clinic location, and participation in the Antihypertensive and Lipid-Lowering Treatment to Prevent Heart Attack Trial (ALLHAT).[15] A data analyst assigned a uniform random number to each of the possible allocations using SAS version 9.1.3 (SAS Institute) and selected the one with the highest random number. At the 6 hospitals randomized to receive a practice-level incentive, physicians could invite up to 15 nonphysician colleagues (eg, nurses, pharmacists) to participate as part of their practice team caring for hypertensive patients on their panel. Also, to meet the physician recruitment goal of 7 per site (to account for attrition), we enrolled additional eligible physicians after randomization. The study was approved by the institutional review boards of all participating institutions.[16] All participants provided written informed consent.

Interventions

Education

Participants attended webinars beginning in February 2008 that reviewed the guidelines from the "Seventh Report of the Joint National Committee on Prevention, Detection, Evaluation, and Treatment of High Blood Pressure (JNC 7)."[17] Participants were also informed of their study group assignments, study measures, and financial incentive amounts.

Financial Incentives

The intervention phase included a 4-month performance baseline period (August-November 2007) and 4 consecutive 4-month periods starting in April 2008. After the end of each period, medical record abstractors at the Houston coordinating center who were blinded to the study aims and study group assignments collected data from electronic medical records for 40 patients with hypertension randomly selected from each physician's panel. Participants who were absent for 4 or more weeks during a given 4-month performance period were not eligible to earn rewards for that period. Practices could replace nonphysician participants who withdrew during the study. Intervention group participants received up to 5 incentive payments in their paychecks approximately every 4 months and were notified each time a payment was posted.

Audit and Feedback

Customized audit and feedback reports detailing performance for each period and the next period's performance goals were posted to the study's secure website. After the final feedback report in April 2010, we followed up participants for a 12-month washout period to determine whether the effects of the intervention were sustained after the incentive ended. We then collected data for a 4-month post-washout performance period, May through August 2011.

Study Measures and Rewards

Participants earned incentives for achieving JNC 7 guideline-recommended blood pressure thresholds or appropriately responding to uncontrolled blood pressure (eg, lifestyle recommendation for stage 1 hypertension or guideline-recommended medication adjustment), prescribing guideline-recommended

Downloaded From: http://jama.jamanetwork.com/ by a Department of Veterans Affairs User on 01/22/2014

antihypertensive medications, or both. The directors of the participating hospital regions contributed $250 000 for the incentives. Results from simulations using pilot data and accounting for estimated improvement rates showed a maximum per-record reward of $18.20, $9.10 for each successful measure.

For the practice-level payments, the aggregated earnings of the physician participants were equally distributed between the physician and nonphysician participants in the practice team. Physicians in the combined incentive group received their individual-level performance payment plus their practice-level share. For example, there are 7 physicians and 3 nurses at a site receiving the combined incentive. If we assume each physician meets both study measures for all 40 records reviewed ($9.10 per measure × 2 measures × 40 records × 7 physicians) in this hypothetical case, the practice's total incentive earning is $5096. For the 10-member practice team, the per-member share is $509.60. Each nurse would receive $509.60, and each physician would receive $1237.60 ($509.60 team share plus $728 for a perfect individual performance).

Sample Size

We adapted an algorithm by Donner and Klar[18] to determine study power. As is standard in such trials, the participants were contained within the units of randomization (clusters). In this study, the participants were primary care personnel and the clusters were VA hospitals. We used estimates of the intraclass cluster correlation of 0.39 for appropriate medication and 0.14 for blood pressure control obtained from pilot data. Equal cluster sizes were assumed to obtain a minimum cluster size. The Donner and Klar algorithm calculates a minimum cluster size and number of clusters using an iterative algorithm based on the noncentral t-distribution. Application of the algorithm yielded a result of 3 clusters per group and 5 physicians per cluster with a minimum of 40 patients per physician with a 95% significance level and 80% power to detect a difference of 17 percentage points between an intervention and control group for appropriate medication and 15 percentage points for blood pressure control. A target minimum of 7 physicians per cluster was used to account for attrition.[19]

Statistical Analysis

To evaluate the adequacy of randomization, we tested for differences in physician characteristics across the groups using χ^2 tests for binomial variables (or Fisher exact test when cell sizes were <5) and the Kruskal-Wallis rank test for continuous variables. We performed a repeated-measures longitudinal analysis using the hospital as a random effect. The unit of analysis was the physician. The analysis approach was intention to treat; physician performance was evaluated according to the group to which his or her hospital was randomly assigned. We evaluated the effect of incentive type for each outcome: (1) each incentive group vs the control group, (2) individual-level incentive groups vs the control group, and (3) practice-level incentive groups vs the control group. The goal of the analysis was to evaluate the rate of change in the proportion of patients who met the study measures over time for the intervention group physicians.[20] Models were developed independently for each incentive type and each study measure. Using an approach described by Cheng and colleagues,[21] we constructed a maximal model using scientifically relevant covariates selected a priori and then performed backward elimination to delete variables with $P \geq .05$, arriving at our final model. The maximal model allowed us to evaluate both the covariance structure and the list of covariates for inclusion in the final model. Maximal model covariates included characteristics of the hospital where the physician practiced (teaching hospital, ALLHAT site, northern vs southern region of the United States), physician demographic and practice characteristics (sex, race, and years practicing since completing residency), characteristics of the physicians' patients sampled for analysis (mean age, percentage male, percentage black, and percentage diabetic), and whether the physician had reached the ceiling value for the study measure in the baseline period. Also, the models included the covariates of time, the effect of the intervention, and the rate of change of the effect of the intervention over time. In addition to the primary study measures, we evaluated whether the physician made any medication adjustment (ie, not solely guideline-appropriate changes).

Because the patients for analysis were randomly selected from a physician's panel at each time point, time-dependent covariates were used. A ceiling value calculated using the Achievable Benchmarks of Care methodology[22] was included in the maximal models because we expected performance to "top off" at some value before reaching 100%. Ceiling values were 95.0% for blood pressure control or appropriate response to uncontrolled blood pressure and 78.3% for guideline-recommended medications. We also determined whether any site-to-site variation existed and included the facility (cluster) as a random effect as necessary. We evaluated final models using a 2-sided *t* statistic with 95% significance and adjusted for multiple comparisons using the Benjamini-Yekutiele method.[23] As a check against overfitting, we conducted a bootstrap analysis in which each physician's patients were resampled and the analysis was repeated 1000 times.

We tracked participants' engagement with the study website. We compared viewing of the baseline or the first intervention period's audit and feedback report between intervention and control group participants using Fisher exact test.

To evaluate performance following the washout, we performed a linear analysis with clustering by hospital with the post-washout performance rate as the dependent variable and the final intervention performance rate as a covariate. We evaluated the effect of each type of incentive and developed the models independently using backward elimination.

Using data from automated processing of structured fields from electronic health records, we evaluated the incidence of hypotension among all patients with hypertension who had at least 1 primary care encounter between February and May 2009. We looked 4 months from the patient's encounter to identify low systolic blood pressure readings (defined as an outpatient systolic blood pressure <90 mm Hg), an outpatient diagnosis of hypotension, or both. All statistical analyses were performed using SAS software version 9.3.

Downloaded From: http://jama.jamanetwork.com/ by a Department of Veterans Affairs User on 01/22/2014

Figure 1. Participant Enrollment

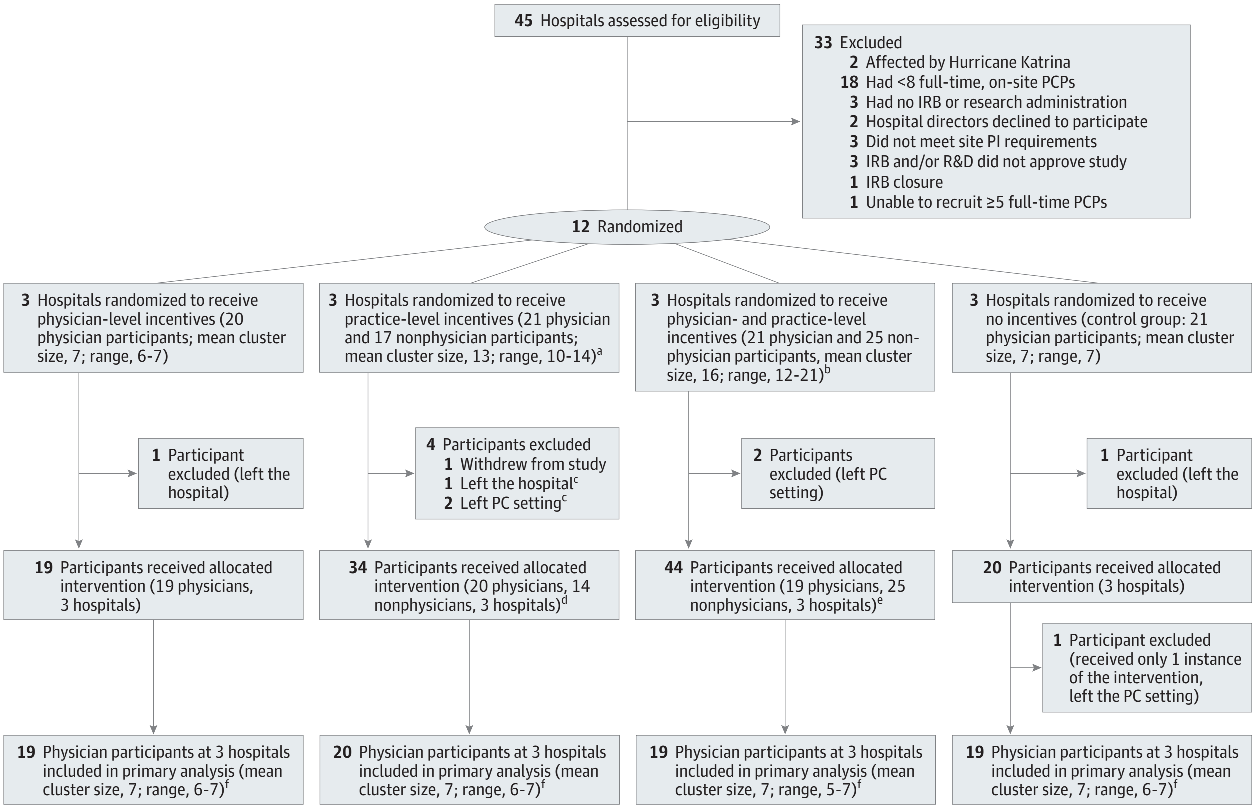

IRB indicates institutional review board; PC, primary care; PCP, primary care practitioners; PI, principal investigator; R&D, research and development.

[a] Nonphysician participants included 7 registered nurses, 4 nurse practitioners, 4 registered nurse case managers, 1 physician assistant, and 1 pharmacist.

[b] Nonphysician participants included 10 licensed practical nurses, 9 registered nurses, 3 medical support assistants, 2 registered nurse care coordinators, and 1 pharmacist.

[c] Nonphysician participant.

[d] Two additional nonphysicians left the practice-level incentives group during the intervention. One new nonphysician was enrolled.

[e] Seven nonphysicians left the physician- and practice-level incentives group during the intervention. Two new nonphysicians were enrolled.

[f] Only data from physicians were used to assess both physician-level and practice-level performances; each physician participant received ≥2 instances of the intervention.

Results

Participant and Site Characteristics

Between February 2007 and April 2008, 83 VA primary care physicians and 42 nonphysician members of practice teams (eg, nurses, pharmacists) were enrolled from 12 study sites (**Figure 1**). Feedback reports displaying participants' baseline performances were provided starting in October 2008. Eight participants withdrew before the first period's feedback report and payment; 7 left the primary care setting or their facility, and 1 withdrew for a personal reason. After participants received intervention components for the first performance period, an additional physician left the primary care setting. Nine nonphysicians (eg, nurses, pharmacists) from 4 sites left during the intervention period and 3 new nonphysicians (eg, nurses, pharmacists) were enrolled. The 77 physicians who contributed at least 2 periods of performance data were included in the analysis. We completed post-washout data collection in April 2012 for the 55 physicians who remained enrolled by the end of the washout period.

Among physicians who participated in all 5 performance periods, the mean (SD) total payment for physicians over the course of the study was $4270 ($459) in the combined group, $2672 ($153) in the individual group, and $1648 ($248) in the practice group. There were no significant differences in the distributions of physician sex, race, years practicing since completing residency, or patient characteristics (**Table 1**). There were significant differences across groups in characteristics of the hospitals where the participants worked, including whether they were teaching hospitals ($P < .001$), whether they were ALLHAT sites ($P < .001$), and whether they were in the southern or northern United States ($P = .04$).

Rewarded Clinical Measures

In unadjusted analyses, the percentage of patients either with controlled hypertension or receiving an appropriate response increased for each incentive group between baseline and the final performance period, 75% to 84% in the individual group, 80% to 85% in the practice group, and 79% to 88% in the combined group. Performance did not change in the control group, 86% (**Figure 2**A). The adjusted estimated ab-

Downloaded From: http://jama.jamanetwork.com/ by a Department of Veterans Affairs User on 01/22/2014

solute change over the study of the patients meeting the combined blood pressure or appropriate response measure was 8.84% (95% CI, 4.20% to 11.80%) for the individual group, 3.70% (95% CI, 0.24% to 7.68%) for the practice group, 5.54% (95% CI, 1.92% to 9.52%) for the combined group, and 0.47% (95% CI, −3.12% to 4.04%) for the control group. The adjusted estimated absolute difference over the study in the change between the proportion of the physician's patients achieving blood pressure control or receiving an appropriate response for the individual incentive group and the controls was 8.36%

Table 1. Distribution of Physician Characteristics Across Study Groups[a]

Characteristic	Incentive Group: Individual (n = 19)	Practice (n = 20)	Combined (n = 19)	None (Control) (n = 19)	P Value[b]
Male sex, No. (%)	8 (42)	12 (60)	10 (53)	13 (68)	.41
Race, No. (%)					
White	7 (37)	7 (35)	9 (47)	8 (42)	.86[c]
Black	3 (16)	2 (10)	1 (5)	0	
Asian Indian	5 (26)	5 (25)	6 (32)	5 (26)	
Other	4 (21)	6 (30)	3 (16)	6 (32)	
Years practicing, mean (SD)	12.9 (8.4)	13.3 (8.7)	11.4 (6.8)	13.4 (7.4)	.88
Workplace type, No. (%)					
Teaching hospital	19 (100)	20 (100)	12 (63)	13 (68)	<.001
ALLHAT site	6 (32)	20 (100)	12 (63)	13 (68)	<.001
Southern United States	13 (68)	7 (35)	12 (63)	6 (32)	.04
Percentage of male patients, mean (SD)[d]	91 (23)	98 (3)	96 (4)	93 (23)	.63
Percentage of black patients, median (IQR)[d]	33 (5-43)	41 (9-48)	10 (5-37)	5 (3-43)	.07
Patient age, mean (SD), y	65 (5)	66 (3)	66 (3)	67 (5)	.69
Percentage of patients with diabetes, mean (SD)[d]	42 (11)	39 (8)	41 (7)	38 (9)	.39

Abbreviations: ALLHAT, Antihypertensive and Lipid Lowering Treatment to Prevent Heart Attack Trial; IQR, interquartile range.

[a] Because the patients for analysis were randomly selected at each time point, characteristics of physicians' patient samples in the first intervention period are displayed.

[b] For binomial variables, P values are from a χ^2 test for difference in distribution across study groups (or Fisher exact test if any cell sizes were <5). For continuous variables, P values are from the Kruskal-Wallis rank test.

[c] Because of cell counts <5, racial categories were collapsed into white vs nonwhite for a χ^2 test of differences across study groups (df = 3).

[d] Denominator by study group: 760 patients (Individual); 800 patients (Practice); 764 patients (Combined); and 760 patients (Control).

Figure 2. Unadjusted Proportion of Physicians' Patients Meeting the Rewarded Clinical Measures by Study Group

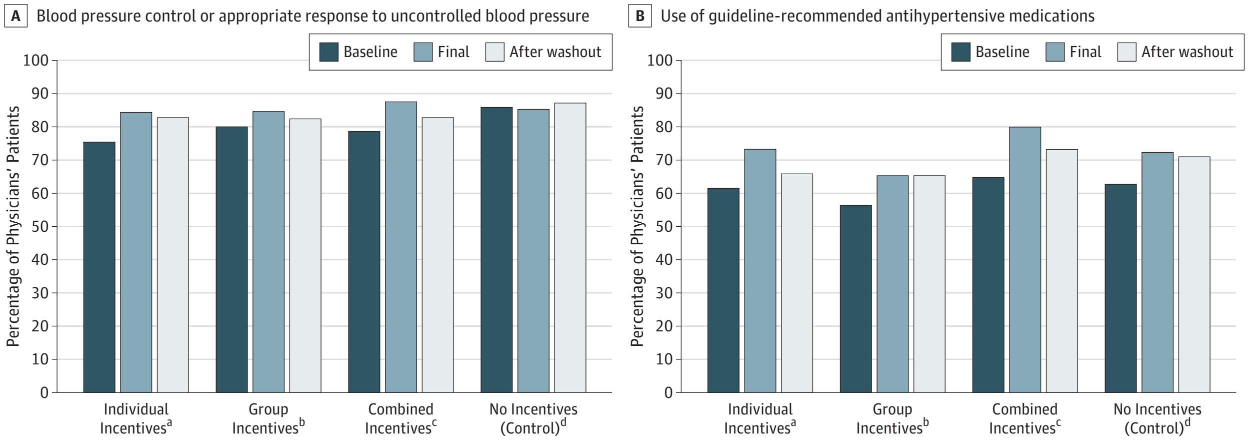

Data are based on review of the electronic medical records of 40 randomly selected patients per physician.

[a]Nineteen physicians participated in the baseline period; 760 patient records reviewed. Eighteen physicians participated in the final intervention period; 720 patient records reviewed. Thirteen physicians participated in the post-washout period; 520 patient records reviewed.

[b]Eighteen physicians participated in the baseline period; 720 patient records reviewed. Nineteen physicians participated in the final intervention period; 760 patient records reviewed. Seventeen physicians participated in the post-washout period; 680 patient records reviewed.

[c]Seventeen physicians participated in the baseline period; 680 patient records reviewed. Fifteen physicians participated in the final intervention period; 600 patient records reviewed. Eleven physicians participated in the post-washout period; 440 patient records reviewed.

[d]Eighteen physicians participated in the baseline period; 720 patient records reviewed. Nineteen physicians participated in the final intervention period; 760 patient records reviewed. Fourteen physicians participated in the post-washout period; 560 patient records reviewed.

Downloaded From: http://jama.jamanetwork.com/ by a Department of Veterans Affairs User on 01/22/2014

Table 2. Longitudinal Modeling Results of the Effect of the Intervention on Rewarded Clinical Measures

	Estimated Change in the Percentage of Physicians' Patients Meeting the Measure, % (95% Bootstrap CI)	Estimated Difference in the Change Between Each Incentive Group and Control Group, % (95% Bootstrap CI)	*P* Value for Estimated Difference[a]
Blood Pressure Control or an Appropriate Response to Uncontrolled Blood Pressure[b]			
Individual incentives	8.84 (4.20 to 11.80)	8.36 (2.40 to 13.00)	.005
Practice incentives	3.70 (0.24 to 7.68)	3.24 (−1.48 to 8.92)	.26
Combined incentives	5.54 (1.92 to 9.52)	5.08 (−0.04 to 10.56)	.09
No incentives (control)	0.47 (−3.12 to 4.04)	1 [Reference]	
Use of Guideline-Recommended Antihypertensive Medications[c]			
Individual incentives	9.07 (4.52 to 13.44)	4.72 (−1.44 to 10.92)	.09
Practice incentives	4.98 (0.64 to 10.08)	0.64 (−5.32 to 7.32)	.81
Combined incentives	7.26 (2.92 to 12.48)	2.92 (−2.76 to 9.64)	.30
No incentives (control)	4.35 (−0.28 to 9.28)	1 [Reference]	

[a] *P* values compare the change in the control group with the change in each incentive intervention group.

[b] Model adjusted for proportion of patients with diabetes, whether the physician worked at a teaching hospital, and whether the physician worked in the northern or southern region of the United States.

[c] Model adjusted for proportion of patients with diabetes, mean age of patients, whether the physician worked at a teaching hospital, and ceiling effects.

(95% CI, 2.40% to 13.00%; *P* = .005; **Table 2**). Thus, a typical study physician in the individual group with a panel size of 1000 patients with hypertension would be expected to have about 84 additional patients achieving blood pressure control or receiving an appropriate response after 1 year of exposure to the intervention. After accounting for multiple comparisons, this remained significant at the .05 level. Significance was confirmed by the bootstrap analysis. Site-to-site variation did not have a significant effect on the modeling results.

Over the course of the trial, unadjusted guideline-recommended medication management increased by the final intervention period compared with baseline in all study groups: 61% to 73% in the individual group, 56% to 65% in the practice group, 65% to 80% in the combined group, and 63% to 72% in the control group (Figure 2B). For the individual, practice, and combined incentive groups and the control group, the adjusted estimated absolute change over the study of the physicians' patients meeting the measure was 9.07% (95% CI, 4.52% to 13.44%), 4.98% (95% CI, 0.64% to 10.08%), 7.26% (95% CI, 2.92% to 12.48%), and 4.35% (95% CI, −0.28% to 9.28%), respectively. Although the use of guideline-recommended medication increased significantly over the course of the study in the intervention groups, there was no significant change compared with controls (Table 2). In adjusted post hoc analyses assessing any medication adjustment (either to start a medication, add a medication, or make a dose adjustment) over the course of the study for the individual incentive group and control group, the absolute difference was 15.36% (95% CI, 0.20% to 28.41%; *P* = .05). For those in the combined incentive group, the difference was 14.80% (95% CI, 0.00% to 27.11%; *P* = .07) (see eTable in the Supplement).

Intervention Fidelity and Unintended Consequences

Far more intervention than control group participants viewed their feedback reports on the website (66 [67%] vs 5 [25%] respectively; *P* = .001), suggesting that participants were aware of the relationship between performance and rewards.

In a model adjusted for geographic region, physician race, and comparison of performance in the final intervention period to the post-washout performance period, there was a significant reduction in performance in the combined measure of blood pressure control or appropriate response to uncontrolled blood pressure in each intervention group compared with controls (**Table 3**). Therefore, the effect of the intervention declined significantly after the incentive was withdrawn.

In post hoc analysis, patients cared for by intervention group participants were no more likely than controls to have hypotension (164 [1.2%] vs 54 [1.4%] patients, respectively; *P* = .18).

Table 3. Linear Regression Analyses of Post-Washout Physician Performance

	Unadjusted Difference in Physician Performance, Estimated Median (IQR)[a]	Difference in the Adjusted Mean Performance Between Each Incentive Group and Control Group, β Coefficient (95% CI)
Blood Pressure Control or an Appropriate Response to Uncontrolled Blood Pressure		
Overall	−2.5 (−7.5 to 5.0)	
By incentive group		
Individual	−2.5 (−7.5 to 2.5)	−9.34 (−14.86 to −3.82)
Practice	−1.3 (−7.5 to 2.5)	−7.14 (−11.87 to −2.42)
Combined	−5.0 (−12.5 to 5.0)	−8.16 (−13.20 to −3.12)
None (control)	0 (−5.0 to 7.5)	1 [Reference][b]
Use of Guideline-Recommended Antihypertensive Medications		
Overall	−5.0 (−10.0 to 2.5)	
By incentive group		
Individual	−10.0 (−12.5 to −5.0)	−10.03 (−16.44 to −3.61)
Practice	−1.3 (−6.3 to 5.0)	−5.60 (−11.25 to 0.04)
Combined	−7.5 (−15.0 to −2.5)	−4.65 (−11.13 to 1.82)
None (control)	0 (−7.5 to 2.5)	1 [Reference][c]

Abbreviation: IQR, interquartile range.

[a] Change in performance from the final intervention period to the post-washout period. Fifty-four of 55 physicians examined for the post-washout period had performance data for both study periods.

[b] Model adjusted for differences in physician performance on the measure in the final intervention period, whether the physician was white or black, and whether the physician worked in the northern or southern region of the United States.

[c] Model adjusted for differences in physician performance on the measure in the final intervention period, proportion of male patients, whether the physician worked in the northern or southern region of the United States, and ceiling effects.

Downloaded From: http://jama.jamanetwork.com/ by a Department of Veterans Affairs User on 01/22/2014

Discussion

In this cluster randomized trial, we evaluated the effectiveness of pay for performance in primary care settings for a common chronic condition. We tested incentives targeted at individual physicians or health care practice teams and a combined incentive to both the individual physician and team. We found that physicians who were randomized to the individual incentive group were more likely than controls to improve their treatment of hypertension as measured by achievement of blood pressure control or appropriate response to uncontrolled blood pressure. Thus, a typical study physician in the individual group with a panel size of 1000 patients with hypertension would be expected to have about 84 additional patients achieving blood pressure control or receiving an appropriate response after 1 year of exposure to the intervention. Although the use of guideline-recommended medications increased significantly over the course of the study in the intervention groups, there was no significant change compared with controls. We also showed that those in the individual incentive group were more likely to make antihypertensive medication adjustments in response to uncontrolled blood pressures. Enhancing the face validity of our findings, participants in intervention groups were far more likely than controls to sign into a secure website and view their performance reports. And similar to a report from Kaiser Permanente, we found that the effect of the intervention was not sustained after the incentive was withdrawn.[24] Although concerns about overtreatment have been cited in criticisms of pay-for-performance programs, we did not find a higher incidence of hypotension in the panels of physicians randomized to the incentive groups.

Some might consider the magnitude of the incentives small. The mean individual incentive earnings over the study represented approximately 1.6% of a physician's salary, assuming a mean salary of $168 000.[25] However, the budget that VA administrative leaders allocated for the incentives was a reflection of their "real-world" constraints, enhancing the generalizability of our findings. Also, the final amounts of the incentives were similar to those recently announced by the New York City Health and Hospitals Corporation for primary care physicians for 13 measures, rather than a single, performance measure,[2] meaning that the incentive we used for a single condition (hypertension) was proportionately greater.

What aspects of the design and implementation of our study may have contributed to our findings? First, our measures are meaningful process measures to clinicians. Second, we measured and rewarded actions mostly under the control of physicians and their practice teams.[26] Because blood pressure is not completely under a clinician's control, we rewarded a combined measure of blood pressure control or an appropriate response to an uncontrolled blood pressure (a so-called "tightly linked" measure).[27,28] Third, responding to an abnormal blood pressure is a discrete task, as opposed to complex problem-solving, such as diagnosing the etiology of abdominal pain. Fourth, we rewarded participants for their absolute rather than relative performance, avoiding a tournament or competition; participants received a prespecified financial incentive each time they met a performance measure.[29,30] These aspects of our study enhanced the salience of the incentive rewards.

In addition, we combined clear audit and feedback with an incentive.[13] Monetary incentives might amplify the positive effects of performance feedback reports.[31] Bandura's self-efficacy theory states that incentives work by piquing an individual's interest in a task, leading to greater effort at performing the task and ultimately to an increased sense of self-efficacy.[32] We found that intervention group participants were much more likely than control participants to view their feedback reports, suggesting that those who received financial incentives demonstrated greater interest in their performance than those who received audit and feedback alone.[33] This suggests that incentive-based interventions and audit and feedback interventions could be synergistic. The goal of the incentive is not to coerce individuals into performing the requested task but to increase their interest in their performance of the task, overcoming clinical inertia.[34]

Unexpectedly, performance gains did not hold after a 12-month washout period, during which we avoided prompting or interacting with participants. Although performance did not decline to preintervention levels, the decline was significant. While we speculate that the cessation of performance feedback information may have contributed to the performance decline, further research should elucidate why this phenomenon occurred.

The VA system has a nationwide quality monitoring and assessment program for primary care and chronic conditions and a culture of performance improvement. As reported by Sutton et al,[9] the cultural context of the performance rewards may be a significant contributor to their effectiveness. Although these contextual factors may have enhanced the likelihood that our intervention was effective, the high baseline performance of VA health care physicians with blood pressure control rates of approximately 75%[35] may have created a "ceiling effect," whereby gains in performance were more difficult to achieve than they might be in the non-VA setting. Therefore, the improvements might have been greater in a system where baseline performance was lower.

Given that health care organizations are restructuring to implement the patient-centered medical home,[11] we assessed the effect of rewards to health care practice teams. We hypothesized a priori that incentives to practice teams would be effective, but we did not find significant effects of either the practice team or the combined incentives. Our interviews with participants suggested that the integrity of the team construct may have been impaired at some sites, perhaps dampening the effectiveness of the practice-level as well as the combined incentives. At 2 of the practice-level incentive sites, participants noted that they did not know who else was in their practice. At 1 site, nonphysician practice team members were moved to different clinic locations, preventing them from working as a team. Participants also noted the importance of a team in improving hypertension management yet confirmed that the team structure they were under was rudimentary (prior to the implementation of the VA Patient-Centered Medical Home known as the Patient Aligned Care Team

Downloaded From: http://jama.jamanetwork.com/ by a Department of Veterans Affairs User on 01/22/2014

[PACT]).[36] Thus, it is possible that, had the PACT structure already been in place, the practice-level and combined incentives would have had a greater effect.

Despite concerns that baseline performance and team cohesion might have dampened the effect of the interventions, several aspects of the VA health care delivery system made this an ideal setting to test the effectiveness of the incentives. First, because the VA uses a single payment approach, we eliminated the problem of multiple payers or varying performance measures diluting the effect of the incentive or the performance targets. Second, VA physicians are salaried, ensuring that the rewards were a clear addition to their expected pay. And while the VA is a uniquely well-suited laboratory for this study, because the structure of the VA health care system is similar to other large delivery systems such as Kaiser Permanente and the Department of Defense, our findings are generalizable. Although VA enrollees are overwhelmingly male, there is little reason to believe that would have systematically biased the study findings.

Hypertension is a common chronic condition, affecting approximately 70% of those 65 years and older,[37] requiring careful follow-up, adjustments to medication and lifestyle, effective patient-doctor communication, and treatment plan adherence. Inadequate blood pressure control results in excess cases of coronary artery disease, congestive heart failure, renal insufficiency, peripheral arterial disease, and stroke.[17] Even small reductions in blood pressure translate into significant reduction in risk of morbidity and mortality[17] and in system-wide costs.[38] This trial addresses the needs of policy makers and payers for information about a clinically relevant payment intervention in routine practice. Payment-system interventions are attractive because of their potential scale and reach. However, payment-system interventions are only one piece of the solution to improve management of chronic diseases such as hypertension. More resource-intensive, tailored, patient-level self-management strategies may be needed to truly affect patient outcomes.

Conclusion

Individual financial incentives, but not practice-level or combined incentives, resulted in greater blood pressure control or appropriate response to uncontrolled blood pressure. None of the incentives resulted in greater use of guideline-recommended medications compared with controls. Further research is needed to understand the factors that contributed to these findings.

ARTICLE INFORMATION

Author Affiliations: Health Policy and Quality Program, Michael E. DeBakey Veterans Affairs Medical Center, Health Services Research and Development Center of Excellence, Houston, Texas (Petersen, Simpson, Pietz, Urech, Hysong, Profit, Woodard); Section for Health Services Research, Department of Medicine, Baylor College of Medicine, Houston, Texas (Petersen, Simpson, Pietz, Urech, Hysong, Profit, Woodard); American Heart Association, Southwest Affiliate, Houston, Texas (Simpson); University of Washington, Department of Health Services, Seattle (Conrad); Philip R. Lee Institute for Health Policy Studies and Department of Medicine, University of California, San Francisco (Dudley).

Author Contributions: Dr Petersen had full access to all of the data in the study and takes responsibility for the integrity of the data and the accuracy of the data analysis.
Study concept and design: Petersen, Simpson, Urech, Hysong, Profit, Conrad, Dudley, Woodard.
Acquisition of data: Petersen, Simpson, Woodard.
Analysis and interpretation of data: Petersen, Simpson, Pietz, Profit, Dudley, Woodard.
Drafting of the manuscript: Petersen, Simpson, Urech.
Critical revision of the manuscript for important intellectual content: Petersen, Simpson, Pietz, Urech, Hysong, Profit, Conrad, Dudley, Woodard.
Statistical analysis: Petersen, Simpson, Pietz, Dudley.
Obtained funding: Petersen.
Administrative, technical, or material support: Petersen, Simpson, Urech, Hysong.
Study supervision: Petersen, Woodard.

Conflict of Interest Disclosures: All authors have completed and submitted the ICMJE Form for Disclosure of Potential Conflicts of Interest and none were reported.

Funding/Support: This work is supported in part by the Veterans Affairs (VA) Health Services Research & Development (HSR&D) Investigator-Initiated Research (IIR) program (04-349; Principal Investigator [PI] Laura A. Petersen, MD, MPH), a National Institutes of Health grant (R01 HL079173-01; PI Laura A. Petersen, MD, MPH), the American Recovery and Reinvestment Act of 2010 (National Heart, Lung, and Blood Institute [NHLBI] 1R01HL079173-S2), and the Houston VA HSR&D Center of Excellence HFP90-020; PI Laura A. Petersen, MD, MPH). Dr Petersen was a recipient of the American Heart Association Established Investigator Award (grant 0540043N) and was a Robert Wood Johnson Foundation Generalist Physician Faculty Scholar (grant 045444) at the time that this study was planned and funded. Dr Hysong was a recipient of an NHLBI Investigator Research Supplement to Promote Diversity in Health-Related Research (1R01HL079173-S1) during the early stages of the study and is currently a VA HSR&D Career Development Awardee (CDA 07-0181). Dr Profit's contribution was supported in part by the Eunice Kennedy Shriver National Institute of Child Health and Human Development (1 K23 HD056298-01; PI Jochen Profit, MD, MPH). Dr Conrad is an awardee of the Robert Wood Johnson Foundation Health Care Financing and Organization Program (grant 63214). Dr Dudley is a Robert Wood Johnson Investigator Awardee in Health Policy.

Role of the Sponsor: The funding sources had no role in the design and conduct of the study; collection, management, analysis, and interpretation of the data; preparation, review, or approval of the manuscript; and decision to submit the manuscript for publication.

Oversight Committee: Barry R. Davis, University of Texas School of Public Health, Houston; Joseph Francis, Department of Veterans Affairs, Washington, DC; Gabriel Habib, Michael E. DeBakey Veterans Affairs Medical Center, Houston; Jeffrey Murawsky, VA Great Lakes Health Care System, Westchester, Illinois; Robert A. Petzel, Department of Veterans Affairs, Washington, DC; Brian Reed, Baylor College of Medicine, Houston.

VA Investigators: Jan Basile, Rose Birkmeier, Warren D. Blackburn Jr, Patricia Cioffi, Peter Friedmann, Saib Gappy, Stephen A. Geraci, Nicholas Haddad, Kent A. Kirchner, James Levenson, Steve Orwig, Alan Pawlow, Adam Powell, Shakaib Rehman, Amy W. Smith, Jessie M. Spencer, Vidya Sridharan, Donald Weinshenker.

VA Primary Care Physician Participants: Vijaya Ajjagottu, Jeffrey Austerlitz, Peter Baren, Andree Burnett, Eugene Constantinou, Donald Curran, Raminder Dhadli, Sean Ercan-Fang, Harold Fain, Swapna Gupta, Helen Han, Shireen Haque, Judith Hildebrand, Claudine Johnson, Yelena Kamenker-Orlov, Thomas Kumenda, Fengqi Liu, George Malatinszky, Minnie Martin, Nicholas Masozera, Ramon Matawaran, Raghuram Matta, Ivan Monserrate, Praveena Mungara, Kimberly Olson, Tuukka Ostenso, Radha Rao, Simona Retter, Terrence Shaneyfelt, Lubna Sheikh, Patricia Sullwold, Vanisree Suverna, Adeyinka Taiwo, Oanh Thai, Ishita Thakar, Rachel Wilson Peery.

Disclaimer: The views expressed are solely of the authors and do not necessarily represent those of the authors' institutions.

Additional Contributions: We thank the following members of the study coordinating center at the Michael E. DeBakey VA Medical Center Health Services Research and Development Center of Excellence: Supicha S. Chitwood, MPH, for coordinating the incentive payments; Mark Kuebeler, MS, for managing the data; Meghan Z. Lutschg, BA, for coordinating data collection; and Richard SoRelle, BS, for managing the institutional review board requirements across the study sites.

Downloaded From: http://jama.jamanetwork.com/ by a Department of Veterans Affairs User on 01/22/2014

We also acknowledge the contributions of the trial's oversight committee, the VA study site investigators, and the VA primary care physician participants. None of the individuals mentioned received compensation for their contributions besides their salaries.

REFERENCES

1. Roadmap for implementing value driven healthcare in the traditional Medicare fee-for-service program. Centers for Medicare & Medicaid Services. http://www.cms.gov/Medicare/Quality-Initiatives-Patient-Assessment-Instruments/QualityInitiativesGenInfo/downloads/vbproadmap_oea_1-16_508.pdf. Accessed July 9, 2013.

2. Hartocollis A. New York City ties doctors' income to quality of care. *New York Times*. January 12, 2013:A1. http://www.nytimes.com/2013/01/12/nyregion/new-york-city-hospitals-to-tie-doctors-performance-pay-to-quality-measures.html. Accessed July 9, 2013.

3. Petersen LA, Woodard LD, Urech T, Daw C, Sookanan S. Does pay-for-performance improve the quality of health care? *Ann Intern Med*. 2006;145(4):265-272.

4. Scott A, Sivey P, Ait Ouakrim D, et al. The effect of financial incentives on the quality of health care provided by primary care physicians. *Cochrane Database Syst Rev*. 2011;9(9):CD008451.

5. Houle SK, McAlister FA, Jackevicius CA, Chuck AW, Tsuyuki RT. Does performance-based remuneration for individual health care practitioners affect patient care? a systematic review. *Ann Intern Med*. 2012;157(12):889-899.

6. Lindenauer PK, Remus D, Roman S, et al. Public reporting and pay for performance in hospital quality improvement. *N Engl J Med*. 2007;356(5):486-496.

7. Werner RM, Kolstad JT, Stuart EA, Polsky D. The effect of pay-for-performance in hospitals: lessons for quality improvement. *Health Aff (Millwood)*. 2011;30(4):690-698.

8. Jha AK, Joynt KE, Orav EJ, Epstein AM. The long-term effect of premier pay for performance on patient outcomes. *N Engl J Med*. 2012;366(17):1606-1615.

9. Sutton M, Nikolova S, Boaden R, Lester H, McDonald R, Roland M. Reduced mortality with hospital pay for performance in England. *N Engl J Med*. 2012;367(19):1821-1828.

10. Epstein AM. Will pay for performance improve quality of care? the answer is in the details. *N Engl J Med*. 2012;367(19):1852-1853.

11. Fields D, Leshen E, Patel K. Analysis and commentary: driving quality gains and cost savings through adoption of medical homes. *Health Aff (Millwood)*. 2010;29(5):819-826.

12. Colla CH, Wennberg DE, Meara E, et al. Spending differences associated with the Medicare Physician Group Practice Demonstration. *JAMA*. 2012;308(10):1015-1023.

13. Petersen LA, Urech T, Simpson K, et al. Design, rationale, and baseline characteristics of a cluster randomized controlled trial of pay for performance for hypertension treatment: study protocol. *Implement Sci*. 2011;6:114.

14. Donner A, Klar N. *Design and Analysis of Cluster Randomization Trials in Health Research.* London, England: Arnold; 2000.

15. ALLHAT Officers and Coordinators for the ALLHAT Collaborative Research Group. Major outcomes in high-risk hypertensive patients randomized to angiotensin-converting enzyme inhibitor or calcium channel blocker vs diuretic: the Antihypertensive and Lipid-Lowering Treatment to Prevent Heart Attack Trial (ALLHAT). *JAMA*. 2002;288(23):2981-2997.

16. Petersen LA, Simpson K, Sorelle R, Urech T, Chitwood SS. How variability in the institutional review board review process affects minimal-risk multisite health services research. *Ann Intern Med*. 2012;156(10):728-735.

17. Chobanian AV, Bakris GL, Black HR, et al; National Heart, Lung, and Blood Institute Joint National Committee on Prevention, Detection, Evaluation, and Treatment of High Blood Pressure; National High Blood Pressure Education Program Coordinating Committee. The Seventh Report of the Joint National Committee on Prevention, Detection, Evaluation, and Treatment of High Blood Pressure: the JNC 7 report. *JAMA*. 2003;289(19):2560-2572.

18. Donner A, Klar N. Statistical considerations in the design and analysis of community intervention trials. *J Clin Epidemiol*. 1996;49(4):435-439.

19. Lachin JM. Introduction to sample size determination and power analysis for clinical trials. *Control Clin Trials*. 1981;2(2):93-113.

20. Verbeke G, Molenberghs G. *Linear Mixed Models for Longitudinal Data.* New York, NY: Springer; 2009.

21. Cheng J, Edwards LJ, Maldonado-Molina MM, Komro KA, Muller KE. Real longitudinal data analysis for real people: building a good enough mixed model. *Stat Med*. 2010;29(4):504-520.

22. Weissman NW, Kiefe CI, Allison J, et al. *Achievable Benchmarks of Care (ABC) User Manual.* Birmingham, AL: University of Alabama at Birmingham, Center for Outcomes and Effectiveness Research and Education (COERE); 2001.

23. Benjamini Y, Yekutieli D. The control of the false discovery rate in multiple testing under dependency. *Ann Stat*. 2001;29(4):1165-1188.

24. Lester H, Schmittdiel J, Selby J, et al. The impact of removing financial incentives from clinical quality indicators: longitudinal analysis of four Kaiser Permanente indicators. *BMJ*. 2010;340:c1898.

25. Department of Veterans Affairs. Annual pay ranges for physicians and dentists of the Veterans Health Administration (VHA). *Fed Regist*. 2009;74(151):39734-39736.

26. Kuhn M. *Quality in Primary Care: Economic Approaches to Analysing Quality-Related Physician Behaviour.* London, England: Office of Health Economics; 2003.

27. Kerr EA, Smith DM, Hogan MM, et al. Building a better quality measure: are some patients with "poor quality" actually getting good care? *Med Care*. 2003;41(10):1173-1182.

28. Woodard LD, Petersen LA. Improving the performance of performance measurement. *J Gen Intern Med*. 2010;25(2):100-101.

29. Conrad DA, Perry L. Quality-based financial incentives in health care: can we improve quality by paying for it? *Annu Rev Public Health*. 2009;30:357-371.

30. Frølich A, Talavera JA, Broadhead P, Dudley RA. A behavioral model of clinician responses to incentives to improve quality. *Health Policy*. 2007;80(1):179-193.

31. Bonner SE, Sprinkle GB. The effects of monetary incentives on effort and task performance: theories, evidence, and a framework for research. *Account Organ Soc*. 2002;27:303-345.

32. Bandura A. *Self-Efficacy: The Exercise of Control.* New York, NY: W.H. Freeman & Co; 1997.

33. Hysong SJ, Simpson K, Pietz K, SoRelle R, Broussard Smitham K, Petersen LA. Financial incentives and physician commitment to guideline-recommended hypertension management. *Am J Manag Care*. 2012;18(10):e378-e391.

34. O'Connor PJ. Overcome clinical inertia to control systolic blood pressure. *Arch Intern Med*. 2003;163(22):2677-2678.

35. Fletcher RD, Amdur RL, Kolodner R, et al. Blood pressure control among US veterans: a large multiyear analysis of blood pressure data from the Veterans Administration health data repository. *Circulation*. 2012;125(20):2462-2468.

36. Klein S. *The Veterans Health Administration: Implementing Patient-Centered Medical Homes in the Nation's Largest Integrated Delivery System.* Washington, DC: The Commonwealth Fund; 2011.

37. Centers for Disease Control and Prevention (CDC). Vital signs: prevalence, treatment, and control of hypertension: United States, 1999-2002 and 2005-2008. *MMWR Morb Mortal Wkly Rep*. 2011;60(4):103-108.

38. Thinking outside the pillbox: a system-wide approach to improving patient medication adherence for chronic disease. New England Healthcare Institute. http://www.nehi.net/publications/44/thinking_outside_the_pillbox_a_systemwide_approach_to_improving_patient_medication_adherence_for_chronic_disease. Accessed July 9, 2013.

Downloaded From: http://jama.jamanetwork.com/ by a Department of Veterans Affairs User on 01/22/2014
