# Supplementary material for: Reports of unintended consequences of financial incentives to improve management of hypertension
Source: PLoS One. 2017 Sep 21;12(9):e0184856. doi: 10.1371/journal.pone.0184856 (PMC5608267; doi:10.1371/journal.pone.0184856)
Supplement: S4 File — (DOCX) [file pone.0184856.s004.docx]

# COREQ Reporting Guidelines Checklist

### Reports of Unintended Consequences of Financial Incentives and Other Quality Improvement Activities to Improve Management of Hypertension

| No | Item | Guide questions/description | Addressed in manuscript |
| --- | --- | --- | --- |
| ***Domain 1: Research Team and Reflexivity*** | | | |
| *Personal Characteristics* | | | |
| 1. | Interviewer/facilitator | Which author/s conducted the interview or focus group? | p.9 |
| 2. | Credentials | What were the researcher’s credentials? E.g. PhD, MD | p.9 |
| 3. | Occupation | What was their occupation at the time of the study? | p. 9 |
| 4. | Gender | Was the researcher male or female? | Not relevant for this study |
| 5. | Experience and training | What experience or training did the researcher have? | p. 9 |
| *Relationship with Participants* | | | |
| 6. | Relationship established | Was a relationship established prior to study commencement? | Not relevant for this study |
| 7. | Participant knowledge of the interviewer | What did the participants know about the researcher? e.g. personal goals, reasons for doing the research | Not relevant for this study |
| 8. | Interviewer characteristics | What characteristics were reported about the interviewer/facilitator? e.g. assumptions, reasons and interests in the research topic | p. 9-10 |
| ***Domain 2: Study Design*** | | | |
| *Theoretical Framework* | | | |
| 9. | Methodological orientation and Theory | What methodological orientation was stated to underpin the study? e.g. grounded theory, discourse analysis, ethnography, phenomenology, content analysis | p. 9 |
| *Participant Selection* | | | |
| 10. | Sampling | How were participants selected? e.g. purposive, convenience, consecutive, snowball | p. 9 |
| 11. | Method of approach | How were participants approached? e.g. face-to-face, telephone, mail, email | p. 9 |
| 12. | Sample size | How many participants were in the study? | p. 11 |
| 13. | Non-participation | How many people refused to participate or dropped out? Reasons? | No dropouts |
| *Setting* | | | |
| 14. | Setting of data collection | Where was the data collected? e.g. home, clinic, workplace | pp. 5-6, 9 |
| 15. | Presence of non-participants | Was anyone else present besides the participants and researchers? | Not relevant for this study |
| 16. | Description of sample | What are the important characteristics of the sample? e.g. demographic data, date | p. 8, 11 |
| *Data Collection* | | | |
| 17. | Interview guide | Were questions, prompts, guides provided by the authors? Was it pilot tested? | Appendix B |
| 18. | Repeat interviews | Were repeat interviews carried out? If yes, how many? | No |
| 19. | Audio/visual recording | Did the research use audio or visual recording to collect the data? | p. 9 |
| 20. | Field notes | Were field notes made during and/or after the interview or focus group? | p. 9 |
| 21. | Duration | What was the duration of the interviews or focus group? | p. 9 |
| 22. | Data saturation | Was data saturation discussed? | Not relevant to this study |
| 23. | Transcripts returned | Were transcripts returned to participants for comment and/or correction? | No |
| ***Domain 3: Analysis and Findings*** | | | |
| *Data Analysis* | | | |
| 24. | Number of data coders | How many data coders coded the data? | pp. 9-10 |
| 25. | Description of the coding tree | Did authors provide a description of the coding tree? | p. 10 |
| 26. | Derivation of themes | Were themes identified in advance or derived from the data? | p. 10 |
| 27. | Software | What software, if applicable, was used to manage the data? | Not reported |
| 28. | Participant checking | Did participants provide feedback on the findings? | No. |
| *Reporting* | | | |
| 29. | Quotations presented | Were participant quotations presented to illustrate the themes / findings? Was each quotation identified? e.g. participant number | pp. 12-16 |
| 30. | Data and findings consistent | Was there consistency between the data presented and the findings? | Yes |
| 31. | Clarity of major themes | Were major themes clearly presented in the findings? | Yes |
| 32. | Clarity of minor themes | Is there a description of diverse cases or discussion of minor themes? | Yes |
