# Supplementary material for: Reports of unintended consequences of financial incentives to improve management of hypertension
Source: PLoS One. 2017 Sep 21;12(9):e0184856. doi: 10.1371/journal.pone.0184856 (PMC5608267; doi:10.1371/journal.pone.0184856)
Supplement: S7 File — (PDF) [file pone.0184856.s007.pdf]

✓Compare

Institutional Review Board for Baylor College of Medicine and Affiliated Hospitals

Protocol Number: H-17777
Status: Approved
Initial Submit Date: 7/6/2005
Approval Period: 7/20/2016 - 7/19/2017

Section Aa: Title & PI

A1. Protocol Title

RANDOMIZED CONTROLLED TRIAL (RCT) OF FINANCIAL INCENTIVES TO TRANSLATE ALLHAT INTO PRACTICE

A2. Principal Investigator

Name:	LAURA ANN PETERSEN	Phone:	713-794-8623
Id:	999644	Fax:	713-748-7359
Department:	MEDICINE: HEALTH SRVCS RESEARCH	Email:	laurap@bcm.tmc.edu
Center:		Mail Stn:	2002 Holcombe (VA 152), Houston, Texas 77030

A3. Administrative Contact

Name:	TRACY URECH	Phone:	713-794-8536
Id:	Non-Baylor	Fax:	713-748-7359
		Email:	tracy.urech@va.gov
		Mail Stn:	2002 Holcombe (VA 152), Houston, Texas 77030

A3a. Financial Conflict of Interest

Does the investigator have a financial interest in any non-Baylor sponsor or funding source for this research?
No

Section Ab: General Information

A4. Co-Investigators

Name:	LECHAUNCY WOODARD	Phone:	713-794-8613
Id:	671930	Fax:	713-748-7359
Department:	MEDICINE: HEALTH SRVCS RESEARCH	Email:	lwoodard@bcm.tmc.edu
Center:		Mail Stn:	

Name:	KENNETH C PIETZ	Phone:	713-794-8644
Id:	992411	Fax:	713-748-7359
Department:	MEDICINE: HEALTH SRVCS RESEARCH	Email:	kpietz@bcm.tmc.edu
Center:		Mail Stn:	

Name:	ROBERT PETZEL	Phone:	612-725-1968
Id:	Non-Baylor	Fax:	
Institution:	Department of Veterans Affairs (Network 23)	Email:	robert.petzel@va.gov
Address:	5445 Minnehaha Avenue, 2nd Floor, Minneapolis, MN 55417		

A5. Funding Source:

Organization: UNITED STATES DEPT OF VETERANS AFFAIRS

A6a. Institutions where work will be performed:

Michael E. DeBakey Veterans Affairs Medical Center

A6b. Research will be conducted outside of the United States:

Country:
Facility/Institution:
Contact/Investigator:
Phone Number:

If documentation of assurances has not been sent to the Office of Research, please explain:

A7. Research Category:

A8. Therapeutic Intent

Does this trial have therapeutic intent?

No

Section B: Exempt Request

B. Exempt From IRB Review

Not Applicable

Section C: Background

There is increasing recognition that opportunities to translate scientific knowledge into clinical practice are being missed, and that there are important blocks impeding the application of clinical knowledge and national guidelines such as the Seventh Report of the Joint National Committee (JNC-7) on the Prevention, Detection, Evaluation, and Treatment of High Blood Pressure into improved health care for patients. The JNC-7 which provides an evidenced- based approach for the prevention and management of hypertension is based on the findings from Antihypertensive and Lipid Lowering Treatment to Prevent Heart Attack Trial (ALLHAT). As an example, for patients with uncomplicated hypertension, the guideline-recommended pharmacotherapeutic intervention is a thiazide diuretic, either alone or combined with drugs from other therapeutic classes. Furthermore, the guideline describes specific high-risk conditions that are compelling indications for the use of medications from other antihypertensive therapeutic classes such as angiotensin-converting enzyme (ACE) inhibitors, angiotensin-receptor blockers (ARB), beta-blockers, and calcium channel blockers (CCB). The Director of the Agency of Healthcare Research and Quality has stated, "We regard research findings that are 'lost in translation' as failures." Guidelines for treatment of hypertension have been widely disseminated to expedite the application of effective advances to clinical practice, but guidelines have had limited effects on improving the quality of health care.

As a result of the growing conviction that explicit financial incentives may play a central role in improving health care quality, a number of pay-for-performance programs are being implemented. The United Kingdom contract proposes that up to one third of general practitioners' income will come from achieving quality targets. Several health plans and the Centers for Medicare and Medicaid Services (CMS) have described pay for performance. At least 35 health plans, covering more than 30 million patients, have some kind of program tying doctor bonuses to performance. General Electric Co., Ford Motor Co. and others are expanding a program, "Bridges to Excellence," that pays doctors bonuses for treating diabetes and heart patients according to guidelines. In the next five to ten years, pay-for-performance- based compensation could account for 20% to 30% of what the federal program pays providers. However, according to the Joint Commission on Accreditation of Healthcare Organizations (JCAHO), these programs are "largely untested." It is sensible that paying more money for services that are of higher quality will result in changes in practice, but there are few studies examining this issue.

Studies of physician-level financial incentives to improve the quality of care are rare in the literature. In Great Britain, general practitioners are paid a lump sum if they achieve a 50% rate of cervical cancer screening among the eligible women in their panel,

with a higher payment if they reach 80%. Physicians increased screenings by close to 50% in one study. However, there was no concurrent comparison group, and others have concluded that improvements could not be attributed to changes in the payment system. Fairbrother et al tested incentives for improving pediatric immunization rates. Sixty physicians were randomly assigned to one of three intervention groups or a control group. The interventions were: 1) bonus and feedback ($1,000 for a 20% improvement from baseline, $2,500 for a 40% improvement from baseline, and $5,000 for reaching 80% up-to-date coverage regardless of baseline performance level); 2) FFS and feedback ($5 for each vaccine administered within 30 days of its coming due, and $15 for each visit at which more than one vaccine was due and all due vaccines were given); and, 3) feedback only. The study duration was 12 months with two instances of feedback. The bonus group improved by 25.3% overall, but none of the other groups improved significantly compared with controls. The cash bonus appeared to be a powerful motivator. More than two-thirds of the physicians in the bonus group improved enough to earn a bonus. Only two of the physicians in the enhanced FFS and two in the feedback-only group improved as much. Because the control group increased more than the fee-for-service (FFS) group, this study and others illustrate the importance of using concurrent controls in studies of this topic.

Incentives may be targeted at a practice group or hospital. Hillman et al tested whether a semi-annual bonus between $570 - $1,260 to a practice would increase screening for cancer. Although both the control and intervention groups increased compliance dramatically (by 100% on average), there was no significant difference between groups. The same investigators randomized 49 pediatric practices to bonus payments for performance on several components of well-child visits. Compliance with preventive care improved, but there was again no difference in the intervention and control groups. Probable reasons for the lack of effect, include: 1) the small magnitude of the incentive; 2) the lack of individual feedback; 3) the fact that only 56% of responders at intervention sites reported being aware of the incentive, and; 4) the fact that most physicians participated in many other health insurance plans, diluting the incentive. In a RCT, Roski et al evaluated the effect of financial incentives to improve adherence to smoking cessation guidelines. Identification of patients' tobacco use status improved in the incentive compared with the control arm (14.1% vs. 6.2%, respectively; P<0.01), but rates of advising or assisting smokers to quit did not differ. Bonuses were given after 1 year, possibly decreasing awareness of the program. Lastly, Kouides and colleagues examined the effect of incentives on influenza immunization rates in 54 practices, with the intervention group receiving an additional $0.80 per shot if the practice attained a 70% or greater rate, plus $1.60 per shot if the rate was at least 85%. The incentive group had a higher increase in immunization rates than the control group (10.3% vs. 3.5%, respectively; P<0.05). In summary, possible explanations for the lack of effect or small effect in these studies of group- level incentives may include lack of awareness of the incentives or negligible size of the incentive. Therefore, group- level incentives have not been adequately evaluated.

Financial incentives can be conceptualized as exerting effects at various levels of the health care delivery system. Physicians and patients interact during the medical encounter. The interaction takes place within a local environment such as a physician group or a hospital, and that interaction within the context of the health care payment system itself. The physician-patient dyad is a type of principal-agent relationship from the economic literature on incentive contracts. Principal-agent theory addresses relationships where one individual (here the patient) cannot directly observe or know the level of skill or effort expended by the other (here the physician) doing the contracted work. Because patients do not have perfect knowledge of their medical condition, patients are willing to have physicians act as their agents. Because patients have asymmetric information about the need for and outcomes of health care, patient demand for health care may be unresponsive to technical quality. Therefore, one advantage of performance pay is that explicit financial incentives are provided even when patient demand for health care is unresponsive to quality. In other words, physician effort in providing high quality is rewarded, regardless of whether patients (or payers) recognize it. Under principal-agent theory, one could anticipate that with hospital-based incentives, physicians cannot acquire the full returns on their individual effort. Thus, they may not provide their full effort. On the other hand, the problem with rewarding physicians and not groups or hospitals (the organizations within which individual physicians practice) is that the required institutional co-operation may not be present, implying that incentives are missing for an important element of the team delivering health care. The role of physicians as agents of two principals (both the patient and the payer) with diverging interests (high quality vs. cost-effective quality) creates challenges in the structure of incentives.

It is important to note that financial incentives and the health care payment system have an important, though not exclusive, influence on the provision of quality. In economic terms, physicians are viewed as maximizing their utility function. (Utility can be defined as well-being.) This function includes, besides income, their professional and social status, altruistic concerns, the cost of the effort to provide the care, and the uncertainty of the clinical effectiveness of treatment. Thus, physicians are affected by non-monetary factors (i.e., personal ethics, professional norms, regulatory control, and clinical uncertainty) and financial incentives (from the payment system). These all affect the physician's effort. This proposal will affect practice by addressing monetary incentives, professional norms (through audit and feedback to providers), and clinical uncertainty by providing an evidence-based recommendation for treatment. Other aspects of the utility function, such as altruism, are important, but beyond the scope of this work.

Paying more for health care services that we know improve quality could have a tremendous impact on improving translation of research findings into health care. The concept of modifying behavior to align incentives of the agent (the doctor) with a principal is not new. Indeed, pharmaceutical companies have been using financial incentives to change physician behavior with great success

for decades. However, as summarized above, the concept of using financial incentives for quality has not been effectively tested. It is reasonable to suppose that the perverse incentives for under-providing quality could be addressed by essentially creating a "fee-for-quality" incentive. However, despite great potential, numerous questions are unanswered. How effective (and cost-effective) are financial incentives for quality? What are the optimum magnitude, frequency, and duration of financial incentives for quality? Can we expect that the effect of financial incentives may persist after they are stopped? Because any effective intervention will have some unanticipated effects, will important patient care activities that are not rewarded financially be neglected? Thus, despite enthusiasm about the potential for aligning financial incentives with high quality health care, there are a number of fundamental unanswered questions about their optimal design, effectiveness, and implementation. Definitive work in assessing the effectiveness of financial incentives for improving quality is non-existent or inadequately evaluated, necessitating this study.

Section D: Purpose and Objectives

(1) To determine the effect of physician-level financial incentives to promote the provision of guideline-recommended hypertension care for outpatients with hypertension; (2) To determine the effect of group-level financial incentives to promote the provision of guideline-recommended hypertension care for outpatients with hypertension; (3) To determine whether there is an additive effect of physician- and group-level financial incentives to promote the provision of guideline-recommended hypertension care for outpatients with hypertension; (4) To determine the persistence of the effect of financial incentives after the intervention ceases; (5) To assess whether there are negative impacts of financial incentives for quality on patients; (6) To assess whether there are negative impacts of financial incentives for quality on the health care organization and delivery system; (7) To determine the relative cost-effectiveness of providing physician-level financial incentives to improve the provision of guideline-recommended hypertension care compared to costs of controls; and (8) To examine the mediating effects of three variables documented in the industrial/organizational psychology literature that significantly affect team performance and thus mediate the effect of the financial incentive: goal commitment, task accomplishment and team interaction shared mental models (SMMs), and planning and strategy development; and (9) To assess the feasibility of the implementation of this project within the VA using electronic databases.

Section E: Protocol Risks/Subjects

E1. Risk Category

Category 1: Research not involving greater than minimum risk.

E2. Subjects

Gender:

Both

Age:

Adult (18-64 yrs)

Ethnicity:

All Ethnicities

Primary Language:

English

Groups to be recruited will include:

Healthy, non-patient, normals

Vulnerable populations to be recruited as subjects:

Employees or lab personnel

Vulnerable populations require special protections. How will you obtain informed consent, protect subject confidentiality, and prevent undue coercion?

The study subjects are VA primary care physicians employed to work as full-time eight-eighth’s (8/8th’s) and full-time VA non-physicians. The study subjects are full-time VA primary care physicians. After subjects from each site have been identified, the informed consent document will be sent to them via email from the research coordinating center in the Houston Center for Quality of Care and Utilization Studies (a VA HSR&D Center of Excellence). The email will include instructions to print the informed consent document and contact a designated staff member of Dr. Petersen's research team via phone at the coordinating center.

Consenting of non-physician subjects will mirror the process used for physician subjects and begin after all physician subjects have

consented and all sites have been randomized. Physician subjects will be instructed to have all non-physician subjects contact the research staff at the Coordinating Center in Houston to initiate the consent process.

The staff member will read the consent document to the subject and will answer any questions subjects may have. At the conclusion of the consent process, the subject will be instructed to sign the consent document and return it in the self-addressed and stamped envelope provided. Subject confidentiality will be protected and maintained to the fullest extent possible. Only Dr. Petersen, her research staff will be aware of subject participation. Undue coercion will be prevented by informing subjects that participation is strictly voluntary and that decision to participate will not impact their employment status.

To assess whether there are impacts of financial incentives for quality on the health care organization and delivery system (aim 6), in addition to study participants, we will also recruit and consent other key stakeholders involved in performance incentive policies, and organizational leadership for participation in debriefing interviews after the end of the intervention period. These stakeholders will be drawn from a pool of Network and facility-level leadership that may include primary care directors; chiefs of staff; and medical center directors. In order to ascertain how financial incentives impact team culture, we also will recruit and consent for these debriefing sessions primary care personnel (e.g., physicians, nurses) who did not participate in the study but who work at the study sites. Finally, in order to collect data on the cost of the intervention (aim 7), we will recruit and consent a hospital-level human resource personnel for a debriefing session.

The informed consent process for the debriefing interviews is described in Section J2. Subject confidentiality will be protected and maintained to the fullest extent possible. Only Dr. Petersen and her research staff will be aware of subject participation. Undue coercion will be prevented by informing subjects that participation is strictly voluntary and that their decision about participation will not impact their employment status.

E3. Pregnant woman/fetus

Will pregnant women be enrolled in the research?

No

E4. Neonates

Will neonates be enrolled in the research?

No

E5. Children

Will children be enrolled in the research?

No

Section F: Design/Procedure

F1. Design

Select one category that most adequately describes your research:

z.z) ARCHIVED DO NOT USE - Other: Financial Incentives, Physicians, Multi-Center

Discuss the research design including but not limited to such issues as: probability of group assignment, potential for subject to be randomized to placebo group, use of control subjects, etc.

For easier reading and comprehension, “we" denotes Dr. Laura Petersen and her research staff.”

This will be a randomized controlled trial (RCT) of physician- and group-level financial incentives plus audit and feedback to to improve the provision of guideline-recommended hypertension care. There will be four study arms:

1) physician-level financial incentive + audit and feedback 2) group-level financial incentive + audit and feedback; 3) physician- and group-level financial incentives and audit + feedback; and 4) audit and feedback only.

Dr. Petersen’s research staff will stratify hospitals on 3 characteristics that a priori we expect are associated with the response to financial incentives and study measures and outcomes of interest: teaching status, ALLHAT study site, and geographic location. ALLHAT study sites provided training on guideline-recommended care for hypertensive patients. We will randomize hospitals within strata to ensure balance across the four study arms. We will randomize hospitals within strata to ensure balance across the four study arms.

Inclusion Criteria:

VA primary care physicians employed to work as full-time eight-eighth's (8/8th's) and non-physician employees employed as full-time at the 12 study hospitals. Primary care physicians are those that work in VA internal medicine, prime care, and women's clinics. . Primary care physicians should not be participants in a simultaneous research intervention to improve care for hypertensive patients. Examples of non-physicians include physician assistants, nurse practitioners, nurses, pharmacists, nutritionists, and administrative support personnel (i.e., clerks). Non-physician individuals who participate as part of a group must be from the same workplace setting where daily or weekly interaction already occurs.

Participants for the post-intervention debriefing sessions (aims 6 and 7) will be drawn from a pool of Network and facility-level personnel including human resource personnel; primary care directors; chiefs of staff; medical center directors; primary care personnel who participated in the intervention; and primary care personnel who did not participate in the intervention but who worked at the participating site. The human resource personnel must have been working in their position for at least 6 months as of the date of consent to be eligible for a debriefing interview. All other personnel must have been working in their position for at least 6 months during the intervention period in order to be eligible for a debriefing interview.

Exclusion Criteria:

VA primary care physicians that do not meet the description listed in the Inclusion Criteria, (i.e. full-time eight-eighth's). In addition, trainees will be excluded. Groups cannot consist of individuals who do not already work as part of a provider group within a hospital.

F2. Procedure

For easier reading and comprehension, “we" denotes Dr. Laura Petersen and her research staff

After the identification of potential study sites (hospitals), Dr. Petersen will contact the Network director and medical director of each hospital study site to recruit the hospital into the RCT. After a hospital has been recruited, we will apply for IRB approval at the site. Once IRB approval is given, the Director of each hospital will provide a listing of primary care physicians employed to work as full time eight-eighth's (8/8th's) at the site. Dr. Petersen will communicate to the potential study physician subjects the objectives and design of the study by videoconference, or grand rounds, and obtain informed consent.

Consenting of non-physician subjects will mirror the process used for physician subjects and begin after all physician subjects have consented and all sites have been randomized. Physician subjects will be instructed to have all non-physician subjects contact the research staff at the Coordinating Center in Houston to initiate the consent process.

If more than seven (7) subjects were to participate, 7 subjects will be randomly selected to be study participants for each particular study hospital. At the time of consent to participate in the study, subjects will be blinded to their study arm assignment he or she will not know his or her study arm assignment. The four study arms are: (1) physician incentive and audit/feedback; (2) group-level incentive and audit/feedback; (3) physician- and group-level incentives; and (4) audit/feedback only. After all the study sites have been recruited, and all subjects have provided consent each hospital will be randomized to one of the four study arms. The physician subject will provide the following information: VA primary care physician number, age, gender, year graduated from medical school, and whether he or she is board certified, and year last certified. The study physician will be informed of his/her study arm assignment after all study physicians have consented to participate in the study. The non-physician subjects will participate only at the study sites randomized to one of the two group-level arms, and be consented after all sites have been randomized.

While IRB approval and physician recruitment activities are ongoing, the PI and her team will also design data collection procedures and interview and recruit research assistants. The PI will train the research assistants and pilot test the data collection procedures.

The physician subjects in all arms will attend an educational presentation, where they will be provided with the rationale for using guideline-recommended anti-hypertensive medications as well as the current JNC-7 hypertension guidelines. Physician subjects and non-physician subjects randomized to the intervention arms will additionally be given information during this presentation about the magnitude and criteria for the incentives. All participants will be notified at this time about the possibility of being selected for a telephone interview at two time points throughout the study, and they will be given the chance to opt out of this portion of the study. Participants will be informed again about the telephone interviews, and provided with a chance to opt out of this component, at a later date via each participant's preferred mode of contact.

Immediately following this educational presentation, participants will be asked to complete a presentation evaluation survey assessing their knowledge and attitudes about hypertension care, the JNC-7 guidelines, and the use of financial incentives. This survey will contain a cover page informing subjects that the survey is being completed for research purposes and is strictly voluntary. The survey will also state that by completing it, the subject is consenting to take the survey; therefore the survey requires a waiver of documentation to consent. The survey will also instruct subjects to place their Study ID number only on it– this will be the only identifier of any kind in the survey. The survey will involve minimal risk to participants. All questionnaires will be

administered by a study team member and maintained securely at the Houston study site.

Incentives and feedback/audit reports are based on a physician's treatment of their patients with hypertension. Sampling frames will be constructed using VA administrative data files from the Austin Information Technology Center (AITC) and other VA data sources. Using the VA administrative data source Primary Care Management Module (PCMM) to identify the patient panel of each physician subject, we will randomly select the records of patients during each 4-month study interval that meet the hypertension eligibility criteria to examine the level of hypertension care in the four months prior to the beginning of the incentive payout period. Using the primary care physician provider number, a designated member of the research staff will access the VA National Patient Care Database by identifying patients with the ICD-9-CM diagnosis code for hypertension, and patients who had a primary care visit with one of the seven subjects (study physicians) from each site during the time period of interest. After identifying the pool of patients from the NPCD, we will also use the Medicare and Medicaid files from the Veterans Affairs Information Resource Center (VIReC) to determine if these patients had Medicare or Medicaid costs in calendar years 2002-2010. The administrative VIReC Medicare files (200,000 locally and 7,000,000 worldwide) contain information on all veteran users who used Medicare services. This will allow us to accurately and more efficiently identify patients with compelling indications that may affect their treatment regimen for hypertension. Administrative data additionally will assist us in identifying patients of black race to randomly sample for sub-analyses in this population. This is an administrative data analysis; we do not enroll patients as subjects for the study.

After the cohort of patients is refined, we will access their medical records from the study hospital's Computer Patient Record System (CPRS) and the Veterans Health Information Systems and Technology Architecture (VISTA) infrastructure. We will access medical records from the study hospital's Computer Patient Record System (CPRS) and the Veterans Health Information Systems and Technology Architecture (VISTA) infrastructure. Use of guideline-recommended antihypertensive medications among ideal patient candidates for them and the proportion of patients achieving national guideline-recommended blood pressure goals will be the primary dependent variables. We will measure the use of guideline-recommended medications among patients with a visit with a study physician. Data will be collected from medical records at each instance of feedback. We will also collect data on the final months of the study's intervention period following the last instance of feedback.

In addition to assessing use of guideline-recommended anti-hypertensive medications and blood pressure control, we will also evaluate the rate of colorectal cancer screening, LDL cholesterol levels, hemoglobin A1C levels, and use of beta blockers for patients with heart disease whose charts will be reviewed. We will exam these indicators of quality of care to determine if there are negative impacts of the financial incentives.

Audit and feedback to individual subjects and provider groups in all arms at five time points over the 20-month study will be provided via a secure website. Data on goal commitment and shared mental models of hypertension care (i.e., task shared mental models) will also be collected at each instance of feedback in the form of a post-feedback survey (see "F.Procedures attachments 12 10 07.doc" in Section S). The survey will use Hollenbeck's seven-item Likert-type survey scale to measure goal commitment, a widely used scale in organizational settings, with a reported reliability of 0.80. For the task shared mental model, we will use the method recommended by Mathieu and colleagues (2005). This is a well-documented method for collecting quantitative mental model data. Participants will rate the likelihood that JNC-7 guideline-recommended components of hypertension treatment (such as lifestyle modification, thiazide diuretics for stage 1 HTN patients, and adding a second medication for stage 2 HTN patients) will effectively manage their patients' hypertension.

Data on comorbid conditions, treatment, medications, lab values, and blood pressure for three of the four prior months will be collected at each data collection point. All subjects, including provider groups will be given information on baseline performance and the Achievable Benchmark of Care. Direct payments to the physicians in the incentive arms will be made every 4 months during the 20 months of the study intervention period. For each instance of feedback, those randomized to the intervention arms will be eligible to receive financial incentives. For those randomized to group-level arms, performance will be measured based solely on patients from physician providers, and not physician's assistants or nurse practitioners. Half of this amount will be based upon the use of guideline-recommended anti-hypertensive medications in ideal candidates. The other half will be based upon the proportion of patients in the physician providers practices' achieving the JNC-7 guideline-recommended blood pressure control targets or who receive appropriate treatment in response to an elevated blood pressure.

Those randomized to the incentive arms will not receive pro-rated incentive payment if they withdraw early from the study. Incentives will be paid every four (4) months over a 20-month period, at five (5) different intervals. They must continue participating through a four-month interval to be considered for incentive pay for that period.

Distribution of the financial incentives will come as a "SPECIAL CONTRIBUTION AWARD." VA Directive 5017* states that, "Network directors are authorized to approve up to and including $7,500 for individual special contribution awards and up to and including $25,000 for group special contribution awards for…title 38 employees (e.g., physicians…)". Network Directors will provide the financial incentives in the study. Therefore, the incentives in this study are in compliance with this Directive.

*Part 1, Appendix B, Pg 1 of http://vaww1.va.gov/ohrm/Directives-Handbooks/Documents/5017a.doc

Using VA administrative data files from the AITC and other VA data sources, including the VA Corporate Data Warehouse, the Decision Support System (DSS), the National Patient Care Database (NPCD), and Fee Basis Files, we will compare the outcomes obtained via these databases to those obtained via chart abstraction in order to assess the feasibility of the implementation of these pay for performance measures within the VA using these data sources.

In order to collect data on the clinic's planning activities and team interaction shared mental models (SMMs), two rounds of telephone interviews will be conducted at each facility, one each at times 2 (study month 8) and 4 (study month 16)

(Due to character limitations, please see attachment "Continuation_F2 Procedures 07 08 2010.doc" in Section S for a continuation of this procedure.)

Section G: Sample Size/Data Analysis

G1. Sample Size

How many subjects (or specimens, or charts) will be used in this study?

Local: 22 Worldwide: 174

Please indicate why you chose the sample size proposed:

The increase in sample size is due to the consenting of non-physician subjects. The non-physician subjects do not have an impact the statistical analysis because the group's performance is being measured based solely on the patients of physicians in the group. Because non-physician subjects' performance cannot be measured at the individual level, their addition to the sample size is only because they must consent to participate and therefore are subjects.

# of Local Subjects # of Subjects at All Sites

Physician Subjects 7 84 Non-Physician Subjects 15 90 Total 22 174

Please note: Non-Physician subjects will participate only at study sites randomized to group-level arms. Therefore, the sample size #'s reflect only 6 sites, and not all sites.

Since thiazide diuretics are the guideline-recommended treatment for first-line pharmacotherapy for patients with uncomplicated hypertension, we provide power calculations for thiazide diuretics. Donner and Klar's method was used to calculate the effect size for various values of the difference in percentage use of thiazide diuretics or blood pressure control we could detect between study arms with 80% power and 95% significance. Using pilot data, Dr. Petersen and her research staff found they could detect a difference of 16 percentage points between the mean proportions in the arms and an effect size of 1.58 for thiazide diuretics with 80% power using a 2-sided t-test at 95% significance with 3 hospitals per study arm and 5 physicians per hospital and sampling 40 patient charts per physician. For blood pressure control, a difference of 16 percentage points can be detected between the mean proportions in the study arms and an effect size of 1.25 with 80% power using a 2-sided t-test with 95% significance with 3 hospitals per study arm and 5 physicians in each arm and sampling 40 patient charts per physician. This sample size must be adjusted to account for anticipated attrition. As described in the application, it is also expected that 5.8% physicians will leave VA employment during the study. Therefore, sample size was inflated by the formula proposed by Lachin to adjust for a drop-out rate of Ro. The unadjusted sample size is multiplied by 1/(1-Ro)2 giving an adjusted sample size of 84 full-time primary care VA staff physicians, or 7 per site (5 physicians + 2 physicians to account for attrition).

In addition to the 40 charts described above, we will also randomly sample additional charts of black hypertensive patents in order to obtain a sufficient number of charts from this minority group that is at higher risk for uncontrolled blood pressure. Although some of the 40 charts described above may be black hypertensive patients, preliminary data reveal that, on average, only about 8 of these will be of black race. In order to detect an increase of 0.20 in the proportion of physicians providing appropriate care to black hypertensive patients due to the intervention with at least 80% power, we would need to sample approximately 18 black hypertensive patient charts per physician. Furthermore, because some physicians will have fewer than 18 such patients in their sampling frame, we will need to oversample from physicians who have more than 18 black hypertensive patients in order to obtain sufficient power. Because the number of black hypertensive patients will vary by provider, and because we will have to weight our analyses accordingly by sampling more charts from physicians who have more such patients, we have not specified a maximum number of charts to sample per provider for this sub-analysis. The outcome measures will be evaluated across study arms (intervention v. control), however, not across providers.

(Due to character limitations, please see "G_sample size attachment_03 10 2010.doc" in Section S for a continuation of this

section)

G2. Data Analysis

Provide a description of your plan for data analysis. State the types of comparisons you plan (e.g. comparison of means, comparison of proportions, regressions, analysis of variance). Which is the PRIMARY comparison/analysis? How will the analyses proposed relate to the primary purposes of your study?

Statistical Analysis for Study Aims 1-5:

Aim 1: To determine the effect of physician-level financial incentives to improve the provision of guideline-recommended care into clinical practice for outpatients with hypertension. Aim 2: To determine the effect of group-level financial incentives to improve the provision of guideline-recommended care for outpatients with hypertension. Aim 3: To determine whether there is an additive effect of physician- and group-level financial incentives to improve the provision of guideline-recommended care for outpatients with hypertension. Aim 4: To determine the persistence of the effect of financial incentives after the intervention ceases. Aim 5: To assess whether there are negative impacts of financial incentives for quality on patients.

Patient-level, physician-level, and hospital-level characteristics will be compared in the control and intervention arms to evaluate the adequacy of randomization. A pre-post intervention analysis will be done for which the outcomes of interest will be the differences in use of guideline-recommended anti-hypertensive medications and blood pressure control. The dependent variable for the analysis will be the proportion of each provider’s patients who are at goal. Of note, patient race will be an important covariate, since patient race may determine response to treatment for hypertension. The equation presented here is for the physician analysis; the group-level analysis will be similar.

The basic model equation for Aims 1 through 5 is: Yij = a0 + a1Xij1 + a2Xij2 + a3Xij3 + a4Xij4 + a5Xij5 + a6Xij6 + a7Xij7 + a7Xij8 + uj + eij where: Yij = proportion of patients for provider i in hospital j who are at goal (i.e., they have been prescribed guideline-recommended medications where appropriate or have controlled blood pressure, or cholesterol level within limits); Xij1 is an indicator variable for the arm, Xij2 is the provider's age; Xij3 is an indicator variable for the provider's gender; Xij4 is the number of years since the provider graduated from medical school; Xij5 =1 if the provider is board certified; 0 if not; Xij6 = 1 if the provider practices at a hospital classified as an academic medical center; 0 if he/she practices at a hospital that is not; Xij7 = 1 if the provider practices at a hospital where ALLHAT training has been provided; 0 if not; Xij8 = the proportion the provider's patients who are at goal at baseline; uj = random effect for the hospital; and eij = random error. Variables obtained from the evaluation survey measuring knowledge and attitudes also will be incorporated into the main model as covariates to account for any potential confounders. The coefficients a0 through a8 are fixed effects. It is necessary for the VAMC to be a random effect because the hospital characteristics would be collinear with a fixed-effect VAMC variable. The risk-adjusted outcomes will be analyzed in a 2-way factorial design, with significance levels corrected for multiple comparisons. The two main effects (physician-level incentive and group-level incentive) and an interaction effect will be tested using standard analysis of variance techniques for two-way factorial designs.

The analysis will be done separately for each of the outcomes. The equation above is for thiazide prescription and cholesterol levels. For blood pressure, three additional variables that describe patient demographics will be added as fixed effects. Since the analysis is at the provider level, the variables will be averaged over the patients measured for each provider. The additional variables are mean age, percent African-American, and percent male.

Randomization will be at the cluster (hospital) level. Adaptive randomization will be used to ensure that hospitals of the same type will not be in the same arm. As pointed out in Donner and Klar (Design and Analysis of Cluster Randomization Trials in Clinical Research, 2000), it is appropriate for the clusters to enter the equation as random effects rather than fixed effects. They also describe how it is advantageous to include the baseline measurement in the equation as fixed effect rather than using the change in proportion from baseline as outcome because this entails fewer assumptions. Finally, Donner and Klar explain how including covariates that are strongly related to the outcome increases power and precision while including covariates that are weakly related to the outcome compromise power and precision. Therefore, for each outcome, we will test each covariate for significance before including it in the final equation.

Aim 6: To assess whether there are negative impacts of financial incentives for quality on the health care organization and delivery system.

Thematic analysis and grounded theory procedures similar to those used for the other telephone interviews previously approved by IRB and already being conducted in this protocol will be conducted by research staff (supervised by our team’s industrial/organizational psychologist) to qualitatively compare differences in unintended consequences and resulting organizational dynamics across the four arms.

Aim 7: To determine the relative cost-effectiveness of providing physician-level financial incentives to improve the provision of

guideline-recommended hypertension care compared to costs of controls.

Cost-effectiveness will be measured with respect to the cost of additional life years saved through the use of financial incentives to achieve guideline-recommended blood pressure goals. Gain in life expectancy has been a suggested measure to standardize the effectiveness of health care interventions. The cost-effectiveness analysis (CEA) will follow the recommendations of the Panel on Cost-Effectiveness in Health and Medicine. The objective of this study is to provide a tool to health decision-makers throughout the VA health care system. Because drug costs are lower in the VA than for most U.S. consumers and third-party payers, a second cost-effectiveness estimate will be made using available data on the Average Wholesale Price of medication; this second estimate may be more generalizable to the U.S. The relevant outcome is the improvement in life expectancy through blood pressure control motivated by the incentive. The cost-effectiveness ratios developed will be cost per physician. Direct costs of the proposed intervention include the costs attributable to increased use of thiazide diuretics due to the incentives, the cost of calculating and disseminating data, and the actual costs of the incentives. Indirect costs are those costs associated with changes in treatments that are associated with use of thiazide diuretics as a complement or substitute, the administrative costs to implement the incentives, and other costs associated with changes in resource usage by hospitals. A decision analysis and Markov model will be performed. An incremental cost-effectiveness analysis will be performed for the competing strategy choices using Decision Analysis Software by TreeAge (DATA). Confidence intervals on the cost-effectiveness ratios will be constructed based on the full range of transition probabilities. The cost-effectiveness analysis (CEA) will be based on the effect of the intervention on transition probabilities between observable states.

The data for the incremental cost calculations will be determined from the Decision Support System (DSS), VA Health Economics Resource Center (HERC) data, VA Pharmacy Benefits Management (PBM) data cost files and the Federal Supply Schedule (FSS) for pharmaceutical costs. Patient-level data will be extracted for patients of the enrolled physicians using using VA administrative data files from the AITC and other VA data sources, including PCMM , CDW, NPCD, DSS, and Fee Basis Files. Both the cost of the physician-patient interaction and the cost of pharmacy will be used. If the incentives invoke a response from physicians, it is likely to increase pharmacy, laboratory, and primary care utilization, compared to the physicians who did not receive the incentive. An average cost per physician will be calculated based on the patients whose records were observed for effect. Administrative costs of the incentive program will be estimated via incremental costs to add study measures to an existing VA quality assessment chart review program called the External Peer Review Program (EPRP). This program operates under the VA Office of Quality and Performance as a contract. EPRP provides diagnostic and procedure-specific quality of care information, permitting internal and external comparisons of clinical care.

The incremental cost-effectiveness ratio (ICER) will be calculated as follows:

ICER = (ACincentive - ACcontrol)/[(BPincentive - BP control) X 1.37] where:AC is the average costs of the incentive and control groups and BP is the number of patients at target blood pressure (BP) control in the incentive and control groups. A sensitivity analysis will be performed to test for sensitivity to various assumptions, including the price of anti-hypertensive medications. Confidence intervals for the ICER will be estimated using a non-parametric bootstrapping approach. Bootstrapping generally outperforms the traditional Taylor series expansion method of confidence interval estimation and has been successfully demonstrated in cost-effectiveness analysis. The bootstrap method estimates the sampling distribution through re-sampling of the collected data. Finally, confidence interval acceptability curves will be developed to quantify the probability that financial incentives are cost-effective relative to maximum cost-effectiveness ratios that would be acceptable to a decision maker using these findings.

Aim 8: To examine the mediating effects of three variables documented in the industrial/organizational psychology literature that significantly affect team performance and thus mediate the effect of the financial incentive: goal commitment, task accomplishment and team interaction shared mental models (SMMs), and planning and strategy development.

The goal commitment and shared mental models survey will be examined with standard mediation analyses as recommended by Baron and Kenny (1986) and more recently by Bauer, Preacher, and Gil (2006). In order to test whether primary care teams engaging in strategic planning will show better performance than those that do not, the telephone interviews at months 8 and 16 will be coded for the frequency and quality of planning efforts, based on Smith & Locke’s dimensions of planning quality. Content analysis and grounded theory procedures similar to those used in previous IRB-approved research conducted by our team’s industrial/organizational psychologist will then be used to qualitatively compare the planning efforts of high and low performing facilities in the group arms.

Aim 9: To assess the feasibility of the implementation of this project within the VA using electronic databases.

Because chart abstracters have the advantage of gaining background and context when they collect data; have access to information in the medical chart not captured in the electronic databases; and participate in re-abstractions of the data that have demonstrated high reliability and validity, the abstracted data is considered to be the “gold standard”. Each measurement obtained using electronic data that does not agree with the chart review data will be flagged as an error. The overall error rate and error

rates by hospital will be computed. Standard normal theory methods will be used to test whether the overall error rate is significantly different from zero, applying the normal approximation to the binomial distribution. Logistic regression with fixed effects will be used to test whether there is a significant difference among facilities.

Section H: Potential Risks/Discomforts

H1. Potential Risks/Discomforts

Describe and assess any potential risks/discomforts and assess the likelihood and seriousness of such risks:

Potential risks and discomforts include the loss of confidentiality for the study participant (physician and non-physician). In addition, for the patients whose charts/records will receive review because their physician is a participant, there is the potential for loss of confidentiality. Although, participants (physicians and non-physicians) will be informed that their decision to participate will not impact or affect their employment status, some participants may still experience feelings of discomfort because the delivery of care they provide to their hypertensive patients will be assessed. We will not provide any performance reports to supervisors.

VISN leadership will not have access to research data. VISN leadership, hospital directors, sections chiefs, department heads, or anyone in a supervisory role will not receive coded de-identified data on physician clinical performance evaluations. Only Dr. Petersen and her research staff at the Houston VA coordinating site will have access to research data and will be responsible for the de-identification process.

H2. Data and safety monitoring plan

Do the study activities impart greater than minimal risk to subjects?

No

H3. Coordination of information among sites for multi-site research

Is the BCM Principal Investigator acting as the SPONSOR-INVESTIGATOR for this multi-site research?

Yes

Is BCM the COORDINATING CENTER for this multi-site research?

Yes

If the answer to EITHER of the questions above is "Yes", please complete the following questions:

If this is a multicenter study and the BCM PI is an INVESTIGATOR with responsibilities of SPONSOR or if BCM is the COORDINATING CENTER, describe the management of information among the sites related to participant protections. Your description should include reporting of unanticipated problems, protocol modifications, IRB and/or institutional approvals, and interim results among the sites.

Each study site (VA hospital) has a site investigator that reports site-specific items (e.g., continuing review requests from the IRB, participant withdrawal) to the project's overall PI (Dr. Petersen) or to study staff members located at the Houston coordinating center who then report these problems to the overall PI. All IRB paperwork (such as protocol modifications, reporting of interim results for a study abstract, and continuing reviews) and participant-related items (such as a participant retired from VA and no longer at the study site) are completed and documented by designated staff at the Houston coordinating center under the direction of the overall PI. All study staff members located at the Houston coordinating center have undergone the required VA human subjects training. Houston coordinating center staff communicate with site investigators and IRB contacts at other sites via electronic mail, encrypted electronic mail, telephone, and secured mail (e.g., Federal Express). The Houston coordinating center is the central repository for the materials related to the study.

None of the remote site investigators have access to the data. All data are collected and analyzed by trained study staff at the Houston coordinating center and secured per VA security guidelines at the Houston Coordinating Center which is housed in the Houston VA HSR&D Center of Excellence (Michael E. DeBakey VAMC). All hard copies of research are kept under double lock and key at the Houston coordinating center. All electronic study data are maintained on the coordinating center's secure server. This server is housed within the Center's computation center, is behind the VA's internal "fire-wall," and has in-house access limited to only those staff involved in data analysis activities.

When remote site investigators are required to present a study binder for a site-specific audit, the materials are sent via secured mail and the investigators are instructed to secure the binder in their office.

When research is conducted in collaboration with outside entities or organizations, the PI must obtain the necessary approvals from

those entities. The BCM IRB may request documentation that such approvals have been obtained. Please list and describe the planned sites for this multi-site research for which the BCM PI is either Sponsor-Investigator and/or Coordinating Center. Sites that do not meet the requirements for inclusion in section A6a of the protocol summary and BCM informed consent documents should be listed here.

In total there are 12 study sites (VA hospitals) participating. Dr. Laura Petersen is the overall PI of the project and the site investigator for the Michael E. DeBakey VAMC site. We have received the necessary approvals at the other sites. The 11 other sites include the Charlie Norwood VAMC in Augusta, Georgia; the Birmingham VAMC; the VA Boston Healthcare System; Ralph H. Johnson VAMC in Charleston, South Carolina; the VA Connecticut Healthcare System, Newington Campus; the John D. Dingell VAMC in Detroit, Michigan; the G.V. Sonny Montgomery VAMC in Jackson, Mississippi; the Minneapolis VAMC; the Oklahoma City VAMC; the Providence VAMC; and the Aleda E. Lutz VAMC in Saginaw, Michigan.

Section I: Potential Benefits

Describe potential benefits to be gained by the individual subject as a result of participating in the planned work.

The participants (physicians and non-physicians) will gain knowledge about improving guideline-recommended hypertension care, and may also learn about VA performance measures and national guidelines. All participants (physician and non-physician) in the study will receive audit and feedback about their patients' use of guideline-recommended antihypertensive medication and blood pressure control as compared to current VA performance standards and JNC-7 guidelines. Provider groups will have the opportunity to learn about their practice patterns and the quality of care the group delivers to their hypertensive patients.

Describe potential benefits to society of the planned work.

The findings from this RCT will provide much needed data about the effectiveness of financial incentives for improving the quality of health care. At this time, many organizations, including Medicare and several commercial plans, are attempting to align financial incentives with the delivery of high quality health care, but there is no evidence to evaluate the outcome of these efforts. This work will add to the literature. Also, this trial will help improve the provision of guideline-recommended hypertension care. It is known that there is a great lag between the translation of scientific knowledge from clinical studies into clinical practice. Also, this work could serve to reshape the financing and delivery of primary care in the VA, possibly influencing health care policy in the VA.

Do anticipated benefits outweigh potential risks? Discuss the risk-to-benefit ratio.

The anticipated benefits greatly outweigh the potential risks. The potential benefits include: providing much needed evidence to answer the question about the effectiveness of pay-for-performance programs; translating information from a clinical study to clinical practice; and educating physicians and provider groups about providing guideline-recommended hypertension care and influencing their care patterns for patients with hypertension (a very prevalent condition that is controlled in less than one-quarter of US citizens).

Section J: Consent Procedures

J1. Waiver of Consent

Will any portion of this research require a waiver of consent and authorization?

No

J1a. Waiver of requirement for written documentation of Consent

Will this research requires a waiver of requirement for written documentation of informed consent?

No

J2. Consent Procedures

Who will recruit subjects for this study?

PI
PI's staff

Describe how research population will be identified, recruitment procedures, and consent procedures in detail.

The research population will be identified, recruited, and consented after each hospital has been recruited into the study. Dr. Petersen and her research staff will submit the protocol for IRB approval at each site. Once IRB approval is granted, the Medical Director from each site will provide research staff with a listing of primary care physicians employed as full-time eight-eighths (8/8th's). Dr. Petersen will communicate the objectives of the study to the potential participants (the primary care physicians) either using videoconferences, or grand rounds. Dr. Petersen will contact eligible physicians in person or via electronic mail or via phone

about their interest in participating in the study. Two documents will be used during the consent process: a) Telephone Script for Consent Process and b) Invitation Letter for MDs.

After physician subjects from each site have been identified, the informed consent document will be sent to them via email. The email will include instructions to print the informed consent document and contact a designated staff member of Dr. Petersen's research team via phone. Research staff will read the consent form to the subject and go over any questions. Research staff will serve as the PI's designee and will document the date and time the consent process took place. After signing it, the subject will return the consent form to Dr. Petersen/research staff. Self-addressed paid postage will be provided to each subject. An individual not involved, engaged, or associated with this research study will serve as the witness during this telephone consent process.

Consenting of non-physician subjects will mirror the process used for physician subjects and begin after all physician subjects have consented and all sites have been randomized. Physician subjects will be instructed to have all non-physician subjects contact the research staff at the Coordinating Center in Houston to initiate the consent process.

Study sites will be randomized to study arms after all physicians have provided their informed consent. Informed consent from non-physician subjects cannot be obtained until all sites have been randomized. Because we will not know which study sites will be randomized to the group-level arms, we have submitted the non-physician consent form to all sites for review so that we have approval for its use at all sites.

The presentation evaluation questionnaire distributed after the JNC-7 educational presentation at study commencement invokes a waiver of documentation to consent subjects. The survey will contain a cover page informing subjects that the survey is being completed for research purposes and is strictly voluntary. The survey will also state that by completing it, the subject is consenting to take the survey. Participants' Study ID number will be the only identifying marker on the survey. The survey will involve minimal risk to participants. All questionnaires will be administered by a study team member and maintained securely at the Houston study site.

The post-feedback questionnaire will be administered to subjects after viewing their performance results on the secure study website. (Due to character limitations, please see attachment "Continuation_J2 Consent Procedures 07 08 2010.doc" in Section S for a continuation of this procedure.)

PLEASE NOTE: This protocol requires the consent (Section Q) of physicians and other VA personnel to participate as subjects. This protocol also requires a justification for waiver of consent and authorization of patients to access their charts/records. Because of this, the justification for waiver of consent and authorization is attached to Section S. Due to character limitation issues, the waiver will not fit within this textbox.

Are foreign language consent forms required for this protocol?

No

J3. Privacy and Intrusiveness

Will the research involve observation or intrusion in situations where the subjects would normally have an expectation of privacy?

No

J4. Children

Will children be enrolled in the research?

No

J5. Neonates

Will non-viable neonates or neonates of uncertain viability be involved in research?

No

J6. Consent Capacity - Adults who lack capacity

Will Adult subjects who lack the capacity to give informed consent be enrolled in the research?

No

J7. Prisoners

Will Prisoners be enrolled in the research?

No

Section K: Research Related Health Information and Confidentiality

Will research data include identifiable subject information?

Yes

Information from health records such as diagnoses, progress notes, medications, lab or radiology findings, etc.

No

Specific information concerning alcohol abuse:

No

Specific information concerning drug abuse:

No

Specific information concerning sickle cell anemia:

No

Specific information concerning HIV:

No

Specific information concerning psychiatry notes:

No

Demographic information (name, D.O.B., age, gender, race, etc.):

Yes

Full Social Security #:

No

Partial Social Security # (Last four digits):

No

Billing or financial records:

No

Photographs, videotapes, and/or audiotapes of you:

Yes

Other:

No

At what institution will the physical research data be kept?

Recording files and any written notes from the telephone interviews at 8 and 16 months and from the post-intervention debriefing sessions will be kept in secure storage. Paper data and recording files will be stored at the VA Houston HSR&D Center of Excellence (Nabisco building; also referred to in the protocol as the ¿Houston Center for Quality of Care and Utilization Studies¿) in Room 121. This room is a locked cabinet storage area.

How will such physical research data be secured?

Physical data will be stored at the VA Houston HSR&D Center of Excellence (Nabisco building; also referred to in the protocol as the Houston Center for Quality of Care and Utilization Studies) in Room 121. This room is a locked cabinet storage area. Access to data with individual identifiers will be restricted. Data for all participants will be identified by study ID number only. Links between the study ID and personal identifying information will be maintained separately in locked storage in the PI's office as per procedures for other studies. Neither the participant's name nor identifying information will be connected to any publications. These data will be maintained at the Houston Center for Quality of Care and Utilization Studies (research site) under the direct supervision of the PI. All research data collected, including identifiers, for this VA research study will be maintained for six years from the date the research study is closed.

At what institution will the electronic research data be kept?

The VIReC Medicare data files are downloaded directly from the VA Central computer center in Austin, Texas (Austin Automation Center [AAC]). All online data will be maintained on the Center's secure UNIX data server with appropriate ID, password, and data access restrictions in place. PCMM data will also be maintained on the Center¿s secure data server. This server is housed within the Center's Computation Center, is behind the VA's internal "fire-wall," and has in-house access limited to only those staff involved

in data analysis activities. Electronic data will be stored on the M drive at the location M:\Research\Petersen_L_Physician Incentive RCT_H-17777 and access to M drive folders is restricted to designated study personnel.

Such electronic research data will be secured via BCM IT Services- provided secured network storage of electronic research data (Non-Portable devices only):

No

Such electronic research data will be secured via Other:

Yes, (describe below):

All online data will be maintained on the Center's secure UNIX data server with appropriate ID, password, and data access restrictions in place. This server is housed within the Center's Computation Center, is behind the VA's internal "fire-wall," and has in-house access limited to only those staff involved in data analysis activities. Study data will not leave the VA. Electronic data will be stored on the M drive at the location M:\Research\Petersen_L_Physician Incentive RCT_H-17777 and access to M drive folders is restricted to designated study personnel. To minimize the risk of loss confidentiality for both the physicians, non-physicians, and their patients whose charts will be reviewed, all members of the research staff will undergo human subject protections training, strict data protocols will be put in place to ensure that only study personnel have access to the data, and data will be retained on secured, password protected hardware. Each patient and study participant will be assigned a unique study ID number. The key linking study IDs and identifiable protected information will be in the possession of only the study PI. There is no plan to disclose or otherwise grant access to VIReC data to entities outside VHA. Only authorized personnel will have access to the data and personnel who no longer need the information will have their access removed. All research data collected, including identifiers, for this VA research study will be maintained for six years from the date the research study is closed.

Will there be anyone besides the PI, the study staff, the IRB and the sponsor, will have access to identifiable research data?

Yes, identify the classes of the persons:

People who ensure quality from the institutions where the research is being done, federal and other regulatory agencies will have access to all of the research data. VISN leadership will not have access to research data. VISN leadership, hospital directors, sections chiefs, department heads, or anyone in a supervisory role will not receive coded de-identified data on physician clinical performance evaluations. Only Dr. Petersen and her research staff at the Houston VA coordinating site will have access to research data and will be responsible for the de-identification process. There is no plan to disclose or otherwise grant access to VIReC data to entities outside VHA. Only authorized personnel will have access to the data and personnel who no longer need the information will have their access removed.

Please describe the methods of transmission of any research data (including PHI, sensitive, and non-sensitive data) to sponsors and/or collaborators.

If the study team is required to transfer sensitive material (e.g., listing of study participants¿ names) to other individuals or entities (e.g., IRBs, VA R&D committees, etc.), the data will either be transmitted electronically through PKI email (or another VA authorized email encryption) or a hard copy will be sent using a secured mailing method such as Federal Express. Any VA data from VA databases used in this study that have to be transferred per a request from a legitimate entity (e.g., VA Office of Research & Development) will be done using an encrypted CD ROM.

Will you obtain a Certificate of Confidentiality for this study?

No

Please further discuss any potential confidentiality issues related to this study.

The greatest confidentiality issue related to this study is securing all information about the participants participating in the study and the records of their patients we are examining. As discussed above all data will be kept in secure storage and accessible by only the PI and the study staff. Data for all participants will be identified by study ID number only.

Section L: Cost/Payment

Delineate clinical procedures from research procedures. Will subject's insurance (or subject) be responsible for research related costs? If so state for which items subject's insurance (or subject) will be responsible (surgery, device, drugs, etc). If appropriate, discuss the availability of financial counseling.

This is not applicable to the study.

If subjects will be paid (money, gift certificates, coupons, etc.) to participate in this research project, please note the total dollar amount (or dollar value amount) and distribution plan (one payment, pro-rated payment, paid upon completion, etc) of the payment.

Dollar Amount:

60

Distribution Plan:

There is no payment given to physicians or non-physicians to participate in the study. Study subjects (physicians and non-physicians) in the intervention arms are eligible for a finanical incentive for performance, not participation.

Books up to a total of $60.00 in value will be given to the individuals that participate in the debriefing interviews. Books will be mailed to the participant's work address.

Section M: Genetics

How would you classify your genetic study?

Discuss the potential for psychological, social, and/or physical harm subsequent to participation in this research. Please discuss, considering the following areas: risks to privacy, confidentiality, insurability, employability, immigration status, paternity status, educational opportunities, or social stigma.

Will subjects be offered any type of genetic education or counseling, and if so, who will provide the education or counseling and under what conditions will it be provided? If there is the possibility that a family's pedigree will be presented or published, please describe how you will protect family member's confidentiality?

Section N: Sample Collection

None

Section O: Drug Studies

Does the research involve the use of ANY drug* or biologic? (*A drug is defined as any substance that is used to elicit a pharmacologic or physiologic response whether it is for treatment or diagnostic purposes)

No

Does the research involve the use of ANY gene transfer agent for human gene transfer research?

No

O1. Current Drugs

Is this study placebo-controlled?

No

Will the research involve a radioactive drug that is not approved by the FDA?

No

Section P: Device Studies

Does this research study involve the use of ANY device?

No

Section Q. Consent Form(s)

None

Section R: Advertisements

None
