# Supplementary material for: Reports of unintended consequences of financial incentives to improve management of hypertension
Source: PLoS One. 2017 Sep 21;12(9):e0184856. doi: 10.1371/journal.pone.0184856 (PMC5608267; doi:10.1371/journal.pone.0184856)
Supplement: S8 File — (DOCX) [file pone.0184856.s008.docx]

# Appendix A: Sample Feedback Report

| **Your Scores This Period**: | **You** | | **Your group** | |
| --- | --- | --- | --- | --- |
|  | # of pts (%) | You earned | # of pts (%) | Group earned |
| Number of eligible hypertensive patients randomly sampled from your panel/group | 40 |  | 280 |  |
| Number of patients who received guideline-recommended BP medications | 36  (90%) | $327.60 | 201  (72%) | $1,829.10 |
| ***Congratulations!*** *You are among the top 10% of performers across the entire study for this period.* | | | | |
| Number of patients with controlled BP | 28  (70%) | $254.80 | 198  (71%) | $1,801.80 |
| Number of patients with uncontrolled BP… | 12  (30%) |  | 82  (29%) |  |
| ... who received an appropriate clinical response to uncontrolled BP this period | 5  (42%) | $45.50 | 40  (49%) | $364.00 |
| **Total Earnings:** | | | | |
| This period - Congratulations! Good work! | $627.90 | | $3,994.90 | |
| Overall | $1,847.30 | | $11,757.20 | |
| **Your Goals for the Upcoming Period:** | **You** | | **Your group** | |
|  | **% of pts.** |  | **% of pts.** |  |
| Percent of eligible hypertensive patients in your panel/group… | | | | |
| … who will receive guideline-recommended BP medications | 90% |  | 85% |  |
| *For the next period, your goal is to maintain or exceed your current performance level. Keep up the great work!* | | | | |
| … with controlled BP | 85% |  | 85% |  |
| … with uncontrolled BP who will receive an appropriate clinical response to uncontrolled BP | 83% |  | 83% |  |
| *If you and your group meet or exceed your goals next period, you and your group could earn at least:* |  | $664.30 |  | $3,985.80 |
| **Total earnings that you will receive this period: $862.89** | | | | |
