# Supplementary material for: Reports of unintended consequences of financial incentives to improve management of hypertension
Source: PLoS One. 2017 Sep 21;12(9):e0184856. doi: 10.1371/journal.pone.0184856 (PMC5608267; doi:10.1371/journal.pone.0184856)
Supplement: S9 File — (DOCX) [file pone.0184856.s009.docx]

# Appendix B: Interview Question Guide

***Unintended Effects***

1. Think about the last routine visit that you had with a patient who had hypertension. Please walk me through that visit.*(possible follow-up probes)*

a. Was this visit with an uncomplicated hypertensive patient or did the patient have many other complications?

b. What about the last time that a patient came in with a problem unrelated to hypertension?

c. Did this study’s focus on hypertension impact your opportunity to deal with other problems important to you or to your patients?

2. How might that visit have gone a year and a half ago?

***Team Dynamics***

3. What about the ways in which you work with your colleagues/staff has changed in the last year and a half?

***Organizational Changes/Impact***

4. Have there been any major changes in your facility/clinic within the last two years that might have impacted the delivery of care to patients *(possible probes/examples: major personnel changes; changes in resources allocated to primary care or the facility; new technology; etc.)*?

***Team Awareness of Participation/Privacy Issues***

5. How closely do you work with any of the other participants in this study?

6. Who *[or who else]* was aware that you were participating in this study?

a. How comfortable would you have been participating in this study if other people knew about your participation?

b. How important is it that your performance information be kept private?

c. To what extent did you feel that was the case with this study?

***Feedback at Facility/Clinic***

7. What kinds of feedback about hypertension care do you receive at your facility?

a. How often do you receive this feedback?

b. Whom does the feedback come from?

c. What data are used to calculate this feedback?

d. How, if at all, has this feedback changed within the last year and a half?

e. What about feedback regarding care for conditions other than hypertension *[repeat questions a-d above if necessary for other conditions]*?

***Perceptions/Attitudes towards Incentives***

8. How do you feel about the idea of providing direct financial incentives to health-care personnel to improve the quality of care? How has that opinion changed during the course of your involvement in this study?

9. Would this incentive help most primary care physicians at your facility to better deliver guideline-recommended care to their hypertensive patients? (possible follow-up: How much of an incentive do you feel would be appropriate?)

***Pay-for-Performance Programs at Facility***

10. Outside of this study, are there any pay-for-performance programs currently in place, or have any ever been implemented at your facility that feature direct financial incentives to health-care personnel for providing higher-quality care to patients?

*If yes:*

a. What was incentivized?

b. How were incentives distributed (ask about amounts)?

c. How were data collected for the incentive program?

d. What was the staff reaction to this incentive program?
