# Supplementary material for: Reports of unintended consequences of financial incentives to improve management of hypertension
Source: PLoS One. 2017 Sep 21;12(9):e0184856. doi: 10.1371/journal.pone.0184856 (PMC5608267; doi:10.1371/journal.pone.0184856)
Supplement: S10 File — (DOCX) [file pone.0184856.s010.docx]

**Appendix C: Table of unintended consequence themes and their associated codes and code definitions**

| Theme | Coded unintended consequence | Code definition |
| --- | --- | --- |
| Patient harm | Access issues | More frequent hypertension care appointments reduced provider time slots. |
|  | Compliance with guidelines harms some patients | Situations in which the rigidity of guidelines may cause harm to patients with specific health needs. |
|  | Deskilling of physicians due to other staff taking over physicians duties | Instances in which elements of hypertension care have been reassigned to another primary care staff member due to new guidelines. |
|  | Incentives negatively impact non-incentivized behaviors | Instances in which the focus on some behaviors, such as hypertension care, reduces the time spent on other care, such as caring for a patient’s diabetes. |
|  | MD too aggressive to the detriment of the patient | Situations in which the incentive’s focus on hypertension may cause a provider to focus too aggressively on the patient’s hypertension to their detriment. |
|  | Patient’s agenda gets crowded out | Situations in which the patient’s primary concerns are dismissed in order to focus on hypertension care. |
|  | Physicians cannot adapt their approach to individual patients | Instances in which the guidelines reduce the provider’s flexibility to treat patients’ cases individually. |
| Documentation | Focus on box-checking/button clicking behaviors | Instances in which the focus becomes on checking boxes or clicking buttons on computer forms rather than actually performing the activities represented by the checkbox/button clicks. |
|  | Excessive time spent on data collection and clinical reminders | Instances in which providers cannot spend enough time focusing on the patient because they are busy collecting requested documentation from the patient. |
|  | Improved documentation without improved care | Situations in which providers note that they have only improved their documentation and have not improved their provision of hypertension care. |
|  | Inaccurate/false documentation | Situations in which a provider notes that someone may enter false information as a means of gaming the system to receive the incentive. |
| Professional morale | Incentive amount deemed insulting | Instances in which an interview participant noted that the incentive amount was low enough to be considered an insult. |
|  | Loss of professional ethos/morality | Instances in which a provider’s motivation for providing hypertension care has shifted towards getting the incentive rather than providing the best care for the patient. |
|  | Lower staff morale | Instances in which the morale of providers or clinical staff not receiving the incentive, or being under-incentivized, may be lowered as a result. |
|  | Other problems with amount of incentive | Other participant complaints about the amount of the incentive. |
|  | Executive/administrative staff rewards deemed unfair | Instances in which problems with incentivizing executive or administrative staff for a provider’s work are discussed. |
| Positive spillover | Incentivizing hypertension care improved non-incentivized care | Instances in which the focus on hypertension care through the incentive improved the provider’s focus on other aspects of care. |
|  | Improved learning and professional development | Instances in which a participant noted that the incentive resulted in a desire to learn more about the care guidelines or hypertension care in general. |
