# Supplementary material for: Reports of unintended consequences of financial incentives to improve management of hypertension
Source: PLoS One. 2017 Sep 21;12(9):e0184856. doi: 10.1371/journal.pone.0184856 (PMC5608267; doi:10.1371/journal.pone.0184856)
Supplement: S3 File — (PDF) [file pone.0184856.s003.pdf]

Hudson, Sonora (HOU)

From: "Jorge Menil" <info@biomedcentral.com> <info@biomedcentral.com>
Sent: Thursday, November 05, 2015 9:18 PM
To: Hudson, Sonora (HOU)
Subject: [EXTERNAL] 00597306 re:permission to reprint table

Dear Dr Hudson

Thank you for contacting BioMed Central.

Reproduction of figures or tables is permitted free of charge and without formal written permission from the publisher or the copyright holder, provided that the figure/table is original, BioMed Central is duly identified as the original publisher, and that proper attribution of authorship and the correct citation details are given as acknowledgement.

If you have any questions please do not hesitate to contact me.

Best wishes

Jorge Menil
Customer Services
info@biomedcentral.com
www.biomedcentral.com
--------------Your Question/Comment -----------------

Greetings,

I am contacting you on behalf of Sylvia Hysong, PhD, and Laura Petersen, MD, MPH, who have sent me a manuscript for editing. They would like to reprint a table in their new manuscript (which will be submitted to a journal other than Implementation Science) that was originally published in one of their manuscripts in Implementation Science. It is Table 1. Study Site Characteristics, from their article, "Design, Rationale, and baseline characteristics of a Cluster Randomized controlled Trial of Pay for Performance for Hypertension Treatment: Study Protocol," by Petersen et al., which was published in Implementation Science 2011;6:114. They will, of course, attribute the original source of publication. Would you please advise the procedure to get permission to reuse this table. We are not sure as to how exactly this works with an open-access journal and were unable to find any information about permissions on your website. Thanks very much for your help. Sonora Hudson, Senior Medical Writer ref:_00D20CUt._50020rzhMtAAI:ref
