# Supplementary material for: Reports of unintended consequences of financial incentives to improve management of hypertension
Source: PLoS One. 2017 Sep 21;12(9):e0184856. doi: 10.1371/journal.pone.0184856 (PMC5608267; doi:10.1371/journal.pone.0184856)
Supplement: S5 File — (PDF) [file pone.0184856.s005.pdf]

June 28, 2006

Baylor College of Medicine
Office of Research
One Baylor Plaza, 600D
Houston, Texas 77030
Phone: (713) 798-6970
Fax: (713) 798-6990
Email: irb@bcm.tmc.edu

LAURA ANN PETERSEN
BAYLOR COLLEGE OF MEDICINE
MEDICINE: HEALTH SRVCS RESEARCH

H-17777 - RANDOMIZED CONTROLLED TRIAL (RCT) OF FINANCIAL INCENTIVES TO TRANSLATE ALLHAT INTO PRACTICE

APPROVAL VALID FROM 6/28/2006 TO 6/6/2007

Dear Dr. PETERSEN

The Institutional Review Board for Human Subject Research for Baylor College of Medicine and Affiliated Hospitals (BCM IRB) is pleased to inform you that the research protocol and consent form(s) named above were approved.

The study may not continue after the approval period without additional IRB review and approval for continuation. You will receive an email renewal reminder notice prior to study expiration; however, it is your responsibility to assure that this study is not conducted beyond the expiration date.

Please be aware that only IRB-approved informed consent forms may be used when written informed consent is required.

Any changes in study or informed consent procedure must receive review and approval prior to implementation unless the change is necessary for the safety of subjects. In addition, you must inform the IRB of adverse events encountered during the study or of any new and significant information that may impact a research participants' safety or willingness to continue in your study.

The BCM IRB is organized and operated according to guidelines of the International Council on Harmonization , the United States Office for Human Research Protections and the United States Code of Federal Regulations and operates under Federal Wide Assurance No. 00000286, issued April 30, 2001. Affiliated hospitals include: the Michael E. Debakey Veterans Affairs Medical Center, St. Luke's Episcopal Hospital, The Methodist Hospital, Texas Childrens Hospital, Texas Institute for Rehabilitation and Research, and the Harris County Hospital District.

Sincerely yours,

GERI L. WOOD,
Institutional Review Board for Baylor College of Medicine and Affiliated Hospitals
